# A novel RANKL‐targeted flavonoid glycoside prevents osteoporosis through inhibiting NFATc1 and reactive oxygen species

**DOI:** 10.1002/ctm2.392

**Published:** 2021-05-21

**Authors:** Guoju Hong, Zhenqiu Chen, Xiaorui Han, Lin Zhou, Fengxiang Pang, Rishana Wu, Yingshan Shen, Xiaoming He, Zhinan Hong, Ziqi Li, Wei He, Qiushi Wei

**Affiliations:** ^1^ Division of Orthopaedic Surgery The University of Alberta Edmonton Alberta Canada; ^2^ Traumatology and Orthopedics Institute Guangzhou University of Chinese Medicine Guangzhou Guangdong P.R. China; ^3^ Department of Orthopaedics The First Affiliated Hospital of Guangzhou University of Chinese Medicine Guangzhou Guangdong P.R. China; ^4^ Division of Bioengineering, School of Medicine South China University of Technology Guangzhou Guangdong P.R. China; ^5^ Department of Endocrinology the Fifth Affiliated Hospital of Guangzhou Medical University Guangzhou Guangdong P.R. China; ^6^ The First Clinical Medical College of Guangzhou University of Chinese Medicine Guangzhou Guangdong P.R. China; ^7^ Department of Orthopaedics The Third Affiliated Hospital of Guangzhou University of Chinese Medicine Guangzhou Guangdong P.R. China

**Keywords:** NFAcT1, osteoclast, osteoporosis, RANKL, Robinin, ROS

## Abstract

**Background and purpose:**

Osteoporosis is characterized by excessive bone resorption due to enhanced osteoclast activation. Stimulation of nuclear factor of activated T cells 1 (NFATc1) and accumulation of reactive oxygen species (ROS) are important mechanisms underlying osteoclastogenesis. Robinin (Rob) is a flavonoid glycoside that has shown anti‐inflammatory and antioxidative effects in previous studies, but little is known about its effects on bone homeostasis. The purpose of our research was to investigate whether Rob could prevent bone resorption in ovariectomized (OVX) mice by suppressing osteoclast production through its underlying mechanisms.

**Methods:**

The docking pose of Rob and RANKL was identified by protein‐ligand molecular docking. Rob was added to bone marrow macrophages (BMMs) stimulated by nuclear factor‐κB (NF‐κB) ligand (RANKL). The effects of Rob on osteoclastic activity were evaluated by positive tartrate resistant acid phosphatase (TRAcP) staining kit and hydroxyapatite resorption assay. RANKL‐induced ROS generation in osteoclasts was detected by H_2_DCFDA and MitoSox Red staining. The classic molecular cascades triggered by RANKL, such as NF‐κB, ROS, calcium oscillations, and NFATc1‐mediated signaling pathways, were investigated using Fluo4 staining, western blot, and quantitative real‐time polymerase chain reaction. In addition, an OVX mouse model mimicking estrogen‐deficient osteoporosis was created to evaluate the therapeutic effects of Rob *in vivo*.

**Results:**

Computational docking results showed that Rob could bind specifically to RANKL's predicted binding sites. *In vitro*, Rob inhibited RANKL‐mediated osteoclastogenesis dose‐dependently without obvious cytotoxicity at low concentrations. We also found that Rob attenuated RANKL‐induced mitochondrial ROS production or enhanced activities of ROS‐scavenging enzymes, and ultimately reduced intracellular ROS levels. Rob abrogated the RANKL‐induced mitogen‐activated protein kinase (MAPK) and NF‐κB signaling pathways, and subsequently blocked NFATc1 signaling and TRAcP expression. In addition, Rob inhibited osteoclast proliferation by downregulating the expression of osteoclast target genes (*Acp5*, *Cathepsin K*, *Atp6v0d2, Nfact1*, *c‐Fos*, and *Mmp9*) and reducing Ca^2+^ oscillations. Our *in vivo* results showed that Rob reduced bone resorption in OVX animal model by repressing osteoclast activity and function.

**Conclusions:**

Rob inhibits the activation of osteoclasts by targeting RANKL and is therefore a potential osteoporosis drug.

## Graphical abstract

In this manuscript, our research is performed to investigate whether Robinin, a novel flavonoid glycoside, could prevent bone resorption in ovariectomized (OVX) mice by suppressing osteoclast production via its underlying mechanisms. Our findings suggest that Rob abrogates the RANKL‐induced MAPK and NF‐κB pathway, attenuates RANKL‐induced reactive oxygen species (ROS) production and subsequently blocks NFATc1 signaling and TRAcP expression. Our *in vivo* results show that Rob reduces bone loss in OVX mice by repressing osteoclast activity and function.

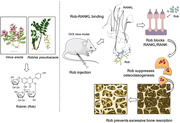

## 1 INTRODUCTION

The regulation of bone formation and resorption mediated by osteoblasts and osteoclasts are complex biological processes. Bone continuously undergoes remodeling, and formation and resorption need to be tightly balanced for maintaining bone homeostasis.^1^ Bone homeostasis disorders are usually caused by osteoporosis.^2^ As the global population ages, osteoporosis is becoming a major economic and social burden, and there is a rising need for effective prevention and treatment strategies.^3^ Therapeutic drugs that inhibit osteoclast activity and differentiation, which may help increase existing bone mass, are considered the first‐line treatment options for osteoporosis.^4^

During osteoclast formation, macrophage progenitor cells (monocytes) fuse to form osteoclasts, which resorb bone tissue.^5^ This process is mainly regulated by two key cytokines: macrophage colony stimulating factor (M‐CSF) and receptor activator of nuclear factor kappa‐B ligand (RANKL).^6^ The interaction between RANKL and RANK directly recruits TNF receptor‐associated factor 6 (TRAF6), and activates series of intracellular molecular events, which involve nuclear factor kappa‐light‐chain‐enhancer of activated B cells (NF‐κB), mitogen‐activated protein kinase (MAPK), and Ca^2+^ pathways, which in turn stimulate the expression of osteoclast‐related downstream factors, such as nuclear factor of activated T cells 1 (NFATc1).^7^ NFATc1 promotes the expression of target factors, including *Tracp*, *Cathepsin K*, and *V‐Atpase D2*, stimulates the maturation of osteoclast precursors, and increases the activities of osteoclast‐related proteins.^8^ Therefore, NFATc1 is considered the key transcription factor in gene expression regulation during osteoclast differentiation, cell fusion, and bone resorption. M‐CSF is also necessary for the proliferation and survival of osteoclast progenitor cells.^9^

Intracellular reactive oxygen species (ROS) accumulation, activated by extracellular RANKL stimulation, plays a pivotal role in the biofunction of osteoclasts.^10^ ROS are produced by osteoclast precursor cells *in vivo* downstream of a signal cascade involving nicotinamide adenine dinucleotide phosphate (NADPH) oxidase 1 (Nox1) and Ras‐related C3 botulinum toxin substrate 1 (Rac1).^11^ For example, a deficiency of Gp91^ph^°^x^, a plasma membrane subunit of NOX1 leads to defects in osteoclast differentiation. This situation is reversed by H_2_O_2_ treatment, suggesting that ROS are an essential factor for osteoclastogenesis.^12^ The protective effects against oxidative stress have been demonstrated for antioxidant enzymes like NADPH oxidase, heme oxygenase‐1 (HO‐1), superoxide dismutase, and various mitochondrial oxidases, which inhibit osteoclastogenesis by promoting cytoprotective enzyme activation.^13^ In RANKL‐mediated signal transduction, the downstream targets of ROS remain unclear, but the increase in oxidative stress may promote the cytoactivity of osteoclasts by triggering the NF‐κB and MAPK signaling pathways.^10^, ^14^, ^15^ Therefore, ROS inhibition is a valuable approach for the osteoporosis therapy.

Robinin (Rob), an active flavone glycoside based on kaempferol, is isolated from *Vinca erecta Regel and Schmalh* or *Robinia pseudoacacia L*.. To date, Rob has shown potential therapeutic effects against cardiac toxicity, inflammation stress, and tumor diseases.^16^, ^17^, ^18^ For example, the highly expressed TLR2 and TLR4, as well as the translocation of NF‐κB p65, induced by the oxidized low‐density lipoprotein were suppressed by the addition of Rob to human peripheral blood mononuclear cells.^17^ Furthermore, a previous study showed that Rob, which has excellent antioxidative properties, also had protective effects on ovine ovarian tissue in vitrification preservation.^19^

Given the important anti‐inflammatory and antioxidative effects of Rob in different applications, we hypothesized that Rob could prevent RANKL‐induced bone loss and osteoclast formation by inhibiting NFATc1 activity, Ca^2+^ ossification, ROS, and NF‐κB/MAPK signaling. Therefore, we analyzed the effects of Rob on (i) RANKL‐induced osteoclast differentiation and the underlying mechanisms in *in vitro* experiments, and (ii) bone matrix loss in an ovariectomized (OVX) animal model *in vivo*.

## 2 MATERIALS AND METHODS

### 2.1 Protein–ligand molecular docking

Rob's 2D structures were obtained from PubChem (Figure S1), while the 3D structure conversion and ligand minimizations were performed using LigPrep of Schrödinger Discovery Suite (Schrödinger, New York, NY, USA). Various possible ionization states were generated at pH 7 ± 2.0, and all states were transferred to the subsequent docking stage. The crystallized structure of the RANKL‐RANK complex was retrieved from PDB database (ID: 4GIQ). The structure was prepared using the Protein Preparation Wizard. The original and optimized structures were compared by Ramachandran Plot (Figure S2), and the optimized structure of RANKL was submitted to the Sitemap for binding site scanning. Next, we generated a receptor grid for the all possible binding sites for the compound docking stage and used in the next stage. During the docking stage, the docking pose of Rob on RANKL with the best‐scoring conformation was obtained through Glide XP docking and docking score comparison. In addition, the effects of Rob's bond with RANKL on protein stability and bond formation were predicted using the Molecular Mechanics‐Generalized Born Surface Area procedure.

### 2.2 *In vitro* osteoclastogenesis assay

An osteoclastogenesis assay was performed using fresh bone marrow macrophages (BMMs). Cells were resuspended and seeded into plates (6 × 10^3^ cells/well), incubated with Alpha Modified Eagle Medium (α‐MEM) supplemented with M‐CSF (50 ng/ml) and recombinant murine sRANK‐ligand protein (rm‐sRANKL, 50 ng/ml), and treated with increasing concentrations of Rob (0, 0.25, 0.5, 1, and 2 μM). The cultured medium was replaced every 2 days to support cell differentiation until multinuclear cells observed. After fixing with 4% paraformaldehyde (PFA), the osteoclast‐like cells were washed with phosphate‐buffered saline (PBS) buffer three times. A TRAcP staining kit (Solarbio Science & Technology Co., Ltd., Beijing, China) was used to examine multinucleate osteoclast‐like cells (nuclei > three). The inhibitory effect of Rob was identified by counting the osteoclast number in each group.

**HIGHLIGHT**

1. Robinin (Rob), an active flavone glycoside based on kaempferol, was isolated from *Vinca erecta Regel and Schmalh* or *Robinia pseudoacacia L*.
2. Computational docking results showed that Rob could bind specifically to RANKL's predicted binding sites.
3. Rob inhibited RANKL‐induced osteoclastogenesis *in vitro*.
4. The *in vivo* results show that Rob reduces loss of bone matrix in OVX mice by repressing osteoclast activity and function.

### 2.3 Cell viability assay

The cytotoxicity of Rob on BMM cells at varying concentrations was measured using an MTS (3‐(4,5‐dimethylthiazol‐2‐yl)‐5‐(3‐carboxymethoxyphenyl)‐2‐(4‐sulfophenyl)‐2H‐tetrazolium) assay kit (Promega Corporation, Madison, WI, USA). Cells were cultured at 6 × 10^3^ cells per well and allowed to sit 12 h in the incubator. After that, the medium was replaced, and different concentrations of Rob (0, 0.25, 0.5, 1, 2, 10, 20, and 30 μM) were added, after which the wells were incubated for 48 h at 37${}^{\circ }C$. Then, α‐MEM supplemented with MTS (20μL) was added to each well, followed by a 2‐h incubation. Cytotoxicity was analyzed using a spectrophotometer (BMG Labtech, Ortenberg, Germany) at 490 nm absorbance.

### 2.4 Hydroxyapatite resorption assay

In order to evaluate the mature osteoclast activities, BMMs were cultured at 1 × 10^5^ cells/well and stimulated with the indicated concentrations of rm‐sRANKL and M‐CSF. If mature cells formed, they were smoothly dissociated and moved to 96‐well Corning osteo assay surface multiple well plates (Corning Inc., Corning, NY, USA) equally in each well. The osteoclasts were cultured in α‐MEM with rm‐sRANKL and M‐CSF in the presence or absence of Rob at 0, 1, and 2M concentrations for 48 h. After that, the wells were separated into two groups for bone resorbed area analysis and cell number counting, respectively. The cells in the first group were removed with bleach. The second group was stained with a TRAcP staining kit as described above. The images in each group were captured by microscopy, and the outcome was obtained from the ratio of resorbed areas to osteoclast number.

### 2.5 Podosome belt assay

In order to observe the podosome belt of RANKL‐induced osteoclasts, BMMs were cultured on fetal bovine serum (FBS)‐coated cover glasses in a 96‐well plate, and cells were treated with Rob (0, 1, or 2 μM) as described above. Cells were fixed in 4% PFA for 8 min, infiltrated with 0.1% (v/v) Triton X‐100 for 10 min, blocked with 3% bovine serum albumin (BSA) for 1 h, incubated 12 h with anti‐vinculin (Sigma‐Aldrich, St. Louis, MO, USA), washed in PBS, and finally cultured with Alexa Fluor 488 (Invitrogen, Waltham, MA, USA). F‐Actin ring was stained with Rhodamine Phalloidin solution (Thermo Fisher Scientific, Waltham, MA, USA) for 1 h. The cells were washed with PBS and subsequently stained with 4,6‐diamidino‐2‐phenylindole (DAPI, Santa Cruz Biotechnology, Dallas, TX, USA), followed by visualization with a confocal microscope (Nikon, Tokyo, Japan).

### 2.6 Flow cytometry

Apoptosis of osteoclasts was evaluated by Annexin V/PI staining of flow cytometry. Specifically, 1 × 10^6^ BMMs were cultured with rm‐sRANKL (50 ng/ml) and M‐CSF (50 ng/ml) for 24 h. Different concentrations of Rob (0, 2, 10, and 20 μM) were then added to treat the cells. Cells were then stained using a Dead Cell Apoptosis Kit (Thermo Fisher Scientific, Waltham, MA, USA) with Annexin V FITC and propidiumiodide (PI). In post‐analysis, cell apoptosis was determined by the following rules (active cells: annexin V‐ and PI‐ viable cells; early apoptotic cells: annexin V+ and PI‐; late apoptotic cells: annexin V+ and PI+; cells die when annexin V‐ and PI+).

Flow cytometry is used to identify the cell cycle of osteoclast differentiation during treatment of Rob. In brief, 1 × 10^6^ BMMs were cultured and stimulated by rm‐sRANKL and M‐CSF as previously described. The next day, the cells were treated with Rob at various dosages of 0, 2, 10, and 20 μM. After fixation, the cells were stained with 10 μg PI as well as ribonuclease (RNase) for half of an hour in a dark place at 37°C, followed by detection using a flow cytometer.

### 2.7 Measurement of ROS levels in osteoclasts

The intracellular ROS levels and mitochondrial superoxide were measured by 2′,7′‐dichlorodihydrofluorescein diacetate (H_2_DCFDA) and MitoSOX Red reagent, respectively. In short, in the presence of Rob (1 or 2 μM), BMMs were stimulated with 50 ng/ml rm‐sRANKL and incubated in 5 mM H_2_DCFDA (Thermo Fisher Scientific, Waltham, MA, USA) for 1 h or loaded with 5 μM MitoSOX Red (Thermo Fisher Scientific, Waltham, MA, USA) for 10 min at 37°C. The fluorescence indicating the ROS activity and mitochondrial superoxide production were detected with a Nikon A1Si confocal microscope at 488 nm (excitation wavelength) and 515–540 nm (emission wavelength). The ratio of average fluorescence intensity as well as the number of ROS‐stained cells was measured in each field by ImageJ.

### 2.8 Intracellular calcium oscillation assay

The calcium oscillation was estimated by a Fluo4‐AM kit (Thermo Fisher Scientific, Waltham, MA, USA). The BMMs seeded at 1 × 10^4^ cells per well were cultured with or without 1 μM Rob in the presence of rm‐sRANKL and M‐CSF for 24 h. The RANKL‐induced osteoclasts were rinsed twice with Hanks' Balanced Salt solution (HBSS) supplemented with 1 mM probenecid as well as 1% FBS, and cultured with 100μL/well Fluo4 solution for 45 min at 37°C. After staining was complete, the cells were washed and kept at room temperature for 20 min. The intracellular free calcium, visualized as fluorescence at different densities, was detected using a fluorescence microscope at 488 mm (excitation wavelength). Images were obtained every 2 s for 1 min. Cells with more than two oscillations were counted as oscillating cells, and their amplitudes were measured using Nikon Basic Research Software.

### 2.9 Quantitative real‐time polymerase chain reaction

Quantitative real‐time polymerase chain reaction (qRT‐PCR) was utilized to evaluate gene expression (*Acp5*, *Cathepsin K*, *Atp6v0d2*, *Nfact1*, *c‐Fos*, and *Mmp9*) during cell differentiation. BMMs were resuspended and cultured with 50 ng/ml M‐CSF and 50 ng/ml rm‐sRANKL at 1 × 10^5^ cells per well in a six‐well plate and pretreated with Rob (0, 0.5, 1, and 2 μM) for 5 days. Total RNA was extracted from the treated cells by Trizol reagent (Life Technologies, Carlsbad, CA, USA). The single‐standard complementary DNA (cDNA) was synthesized from 1μg of RNA template using Moloney murine leukemia virus (M‐MLV) reverse transcriptase with an oligo‐dT primer (Promega Corporation, Madison, WI, USA). qRT‐PCR was performed using a real‐time PCR machine (Applied Biosystems, Warrington, Cheshire, UK). *Gapdh* was used as an internal reference for the gene expression levels. Primers for qRT‐PCR are listed in Table S1.

### 2.10 Measurement of transciptional activities and nulcear translocation

In order to identify NF‐κB and NAFTc1 transcriptional activities, RAW264.7 cells (American Type Culture Collection, Manassas, VA, USA) were stably transfected with either p‐NF‐κB‐TA‐Luc or p‐NAFTc1‐TA‐Luc, which were luciferase reporter constructs responding to NF‐κB and NAFTc1, respectively.^20^, ^21^ After that, the transfected cells were seeded at an equal 1.5 × 10^3^ cells per well to achieve confluence overnight. Then, cells were treated with different concentrations of Rob (0.5, 1, and 2 μM) for 1 h. After pretreatment, the Luc‐NF‐κB cells were incubated with 50 ng/ml rm‐sRANKL for 6 h, while the luc‐NFATc1 cells were incubated for 24 h with the added Rob. Eventually, the luciferase activity of the lysed cells was evaluated by a luciferase reporter assay kit (Promega Corporation, Madison, WI, USA).

In addition, immunofluorescence staining was performed to evaluate the nuclear translocation of p65 (also known as RelA, is one of the five components that form the NF‐κB transcription factor family) and NFATc1. BMMs were seeded into a 12‐well plate with concentration of 13 × 10^4^ cells/well and treated with Rob (1 μM) for 30 min in advance. After that, the cells were stimulated by RANKL and M‐CSF as previously for 30 min. After fixing with 4% PFA, the cells were blocked with 2% bovine serum albumin (diluted by PBS) for 1 h and incubated with anti‐p65 or anti‐NFATc1 antibodies at 4°C for 12 h followed by Alexa Fluor 633 or Dylight 488 secondary antibody for 1 h in the dark. Finally the immunofluorescence images were obtained and merged with DAPI‐stained nuclei images.

### 2.11 Western blot assay

BMMs were cultured onto complete medium supplemented with rm‐sRANKL and M‐CSF, with or without Rob (1 μM) for the indicated time points. Untreated cells served as a negative control group. The cells were then lysed with radioinmunoprecipiation lysis buffer (Millipore, Burlington, MA, USA). In the GTP‐Rac1 evaluation, cell lysis solution was cultured with PAK1 PBD protein (Sigma‐Aldrich, St. Louis, MO, USA) and measured by an active Rac1 Pull‐Down and Detection Kit (Cell Signaling Technology, Danvers, MA, USA). The cellular proteins were isolated using 10% sodium dodecyl sulfate‐polyacrylamide gels, and subsequently moved to polyvinylidene fluoride (PVDF) membranes obtained from GE healthcare (Chicago, IL, USA). The membranes were blocked with 5% skim milk powder for 1 h, after which primary antibodies were administered to blot the membranes at 4${}^{\circ }C$ for 12 h. After that, the membranes were washed and transferred for 1‐h incubation of appropriate secondary antibodies. Finally, immunoreactivity was visualized using enhanced chemiluminescence reagents (PerkinElmer, Waltham, MA, USA), and the membranes were exposed to an Image‐quant LAS 4000 (GE Healthcare, Chicago, IL, USA).

### 2.12 *In vivo* ovariectomy mouse model

The animal protocol was approved by the animal ethics broad where the animal study was performed (Number: 20200328023). An osteoporosis murine model was established to estimate the therapeutic effects of Rob on osteoclast‐mediated bone resorption *in vivo* (Figure 7A). In brief, 36 pathogen‐free C57BL/6J mice (female, 7 weeks old) were randomly assigned to three groups (12 mice for each group; half for toxicity testing and half for bone mass evaluation): a sham group, an ovariectomy (OVX) group, and an OVX+Rob (6 mg/kg) group. After 7 days of acclimatization, mice were anesthetized, and a bilateral OVX operation was performed in the OVX and OVX+Rob groups, while a sham procedure was performed on the normal control mice. After a 1‐week recovery period, the mice in the OVX+Rob group were given a Rob treatment of 6 mg/kg intraperitoneally (optimized concentration confirmed in pre‐experiment, see Figure S3) every other day for 6 weeks. The same volume of vehicle (1% DMSO dissolved in PBS solution) was intraperitoneally injected into the models of the two other groups.

After treatment for 6 weeks, the mice were humanely euthanized. The abdominal aortas of half of the mice in each group were exposed along the midline of the abdomen. Blood samples was drawn from the abdominal aorta of mice and centrifuged to obtain serum for enzyme‐linked immunosorbent assay (ELISA) testing. Serum levels of TRAcP and C‐terminal telopeptide (CTX‐1), indicating osteoclast activity, were estimated using the ELISA kits (R & D company, Minnneapolis, MN, USA). Subsequently, left tibia samples were collected for microstructural testing and right one samples for morphological analysis.

For the other half of the mouse cohort, organ samples of mice from each group were isolated to detect liver, spleen, lung, heart, and renal toxicity by analyzing the size and surface gloss of each organ. Hematoxylin & eosin (H&E) staining was used for the microscopic examination of isolated organs. Blood samples were also extracted from the mouse aortas to analyze complete blood count using a BC6800 automated analyzer (Mindray, Shenzhen, China) in our clinical laboratory.

### 2.13 Micro‐CT scanning

The micro‐architecture of the trabecular bone of the distal tibia was determined using a high‐resolution micro‐CT (Scanco Medical, Wangen‐Brüttisellen, Switzerland). Specifically, after removing soft tissue, trabecular bone from the metaphysical regions of the proximal tibia was assessed using a micro‐CT machine. The analyses were performed using a 60‐kVp X‐ray source voltage, a 500 μA current, 40 W power, an isotropic pixel size of 9 μM, a resolution of 20μm, and an average of 6 frames. Structural parameters in a square region of interest (ROI) set at 0.5 mm from the tibia growth plate were analyzed using the CTAN program (Bruker micro‐CT, Kontich, Belgium), including bone volume/tissue volume (BV/TV), number of trabeculae (Tb. N), connectivity density (Conn.Dn), and thickness of trabeculae (Tb.Th).

### 2.14 Histomorphometric tibia analysis

For histomorphometric analysis of the tibias, the specimens were fixed with 4% PFA and decalcified with 10% ethylenediaminetetraacetic acid soaking solution for another three weeks until soft. The tibias were dehydrated and embedded in paraffin and sectioned using a micro‐tome (4 μm thick). Finally, the sections were stained using a TRAcP kit and hematoxylin & eosin (H&E) to identify the osteoclastic resorption. The addition of outer length of trabecula within ROI was defined as bone surface. The TRAcP‐positive cellular structures were defined as osteoclast‐like cells. The outer length and number of osteoclast‐like cells were calculated. Finally, the quantitative parameter, ratio of osteoclast surface to bone surface (Oc.S/BS), and ratio of amount of osteoclasts to bone surface (N.Oc/BS) were evaluated manually.

### 2.15 Biomechanical properties of the bone

To address the effect of Rob on biomechanical properties of bone, a three‐point bending test was performed. The mouse tibias were placed on two detached basal seats with a load adding on the mid‐point of the tibia. Mechanical resistance to failure (displacement and load applied) was measured using a 5967 Series Universal Testing Systems (Instron, MA, USA) with actuator displaced at 2 mm/min. The ratio of ultimate force (Unit: Newtons [N]) and yield point (N) versus body weight of mice was evaluated according to the instruction.

### 2.16 Materials and reagents

Rob was purchased from ChemFaces (CAS No. 301‐19‐9, catalog number: CFN98375, purity≥98%, Wuhan ChemFaces Biochemical Co., Ltd., Wuhan, China) and dissolved in a concentration of 1 mM nuclease‐free water and DMSO. C57BL/6 mice for the cellular and animal experiments were purchased from the Experimental Animal Center of Guangzhou University of Chinese Medicine. The culture media, α‐MEM, HBSS, and FBS were obtained from Gibco (Carlsbad, CA, USA). Rm‐sRANKL was purchased from PeproTech (Rocky Hill, NJ, USA), and recombinant M‐CSF was obtained from Sigma‐Aldrich (St. Louis, MO, USA). Detailed information regarding the antibodies used is listed in the Table S2.

### 2.17 Statistical analysis

All presented data were shown as means ± standard deviation from multiple independent tests. Each test was performed at least three times. Statistical significance was determined using Student's *t* test, with *p* less than 0.05 regarded as significant.

## 3 RESULTS

### 3.1 Identification of affinity of Rob‐RANKL complex

**FIGURE 1:**
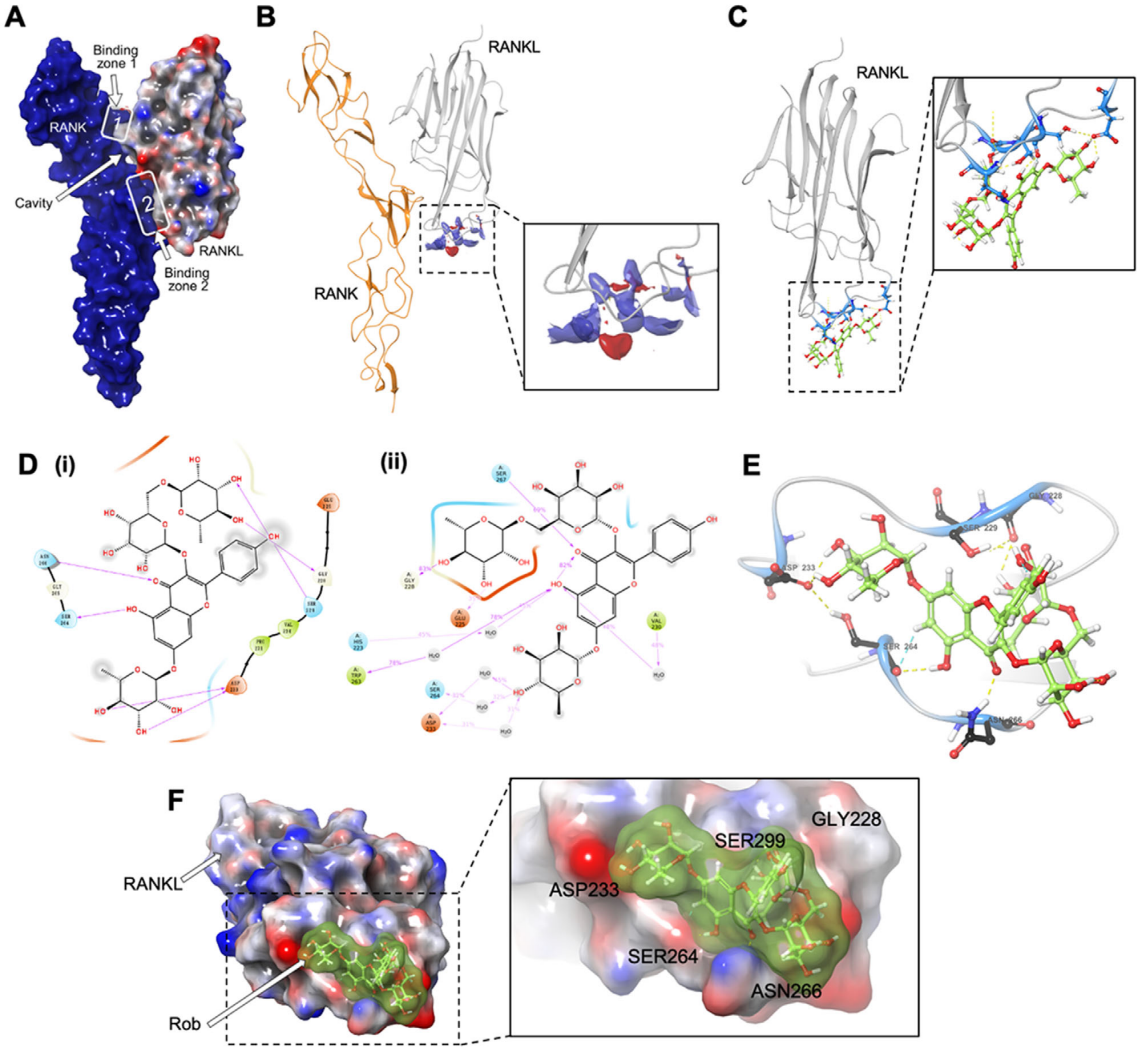
Computational docking result of the Rob‐RANKL interaction. (A) Structural 3D image showing that the RANK/RANKL complex interface is discontinuous, with two binding zones forming a cavity between cytokine and receptor. (B) Image in secondary structure indicating the potential binding sites of RANKL (red region: hydrogen‐bond accepter; blue region: hydrogen‐bond donor; yellow region: hydrophobic domain). (C) Image in secondary structure showing the interaction of RANKL and Rob. The side chains of amino acid residues involved in bonding are marked in blue. (D) Images showing non‐covalent interactions of Rob and RANKL (i) as well as the interaction in mimic liquid environment (ii). (E) Image in secondary structure showing the bonding of Rob and RANKL (blue region: amino acids involved in bonding; green region: Rob; yellow line: hydrogen bonds with polar atoms, blue line: hydrogen bonds with benzene ring π electron cloud). (F) Structural 3D image indicating the non‐covalent interaction between RANKL and Rob in advanced structure. Main amino acids define the edges of the Rob binding groove in RANKL's predicted site.

To define the novel interaction between Rob and RANKL, we developed a multiple‐step procedure to explore the affinities of Rob and RANKL. The crystal model of the RANK‐RANKL complex was established as a basic template for matching Rob in interaction with RANKL (Figure 1A). In accordance with the structural changes in RANK upon RANKL binding, we predicted a potential binding site for Rob in a hydrophobic domain containing hydrogen‐bond accepter and donor (Figure 1B). Specifically, the binding site for Rob docking started from residues ASP233 on RANKL, ran through a binding groove among SER299/GLY288 and SER264, and finally extended downward toward ASN266 (Figures 1C–1F). Computational docking revealed that RANKL had a strong affinity for Rob (Rob binding free energy = ‐7.44 kcal/mol). Taken together, our results revealed that the high affinity between Rob and RANKL was based on their complex non‐covalent interactions.

### 3.2 Rob prevents RANKL‐mediated osteoclastogenesis

**FIGURE 2:**
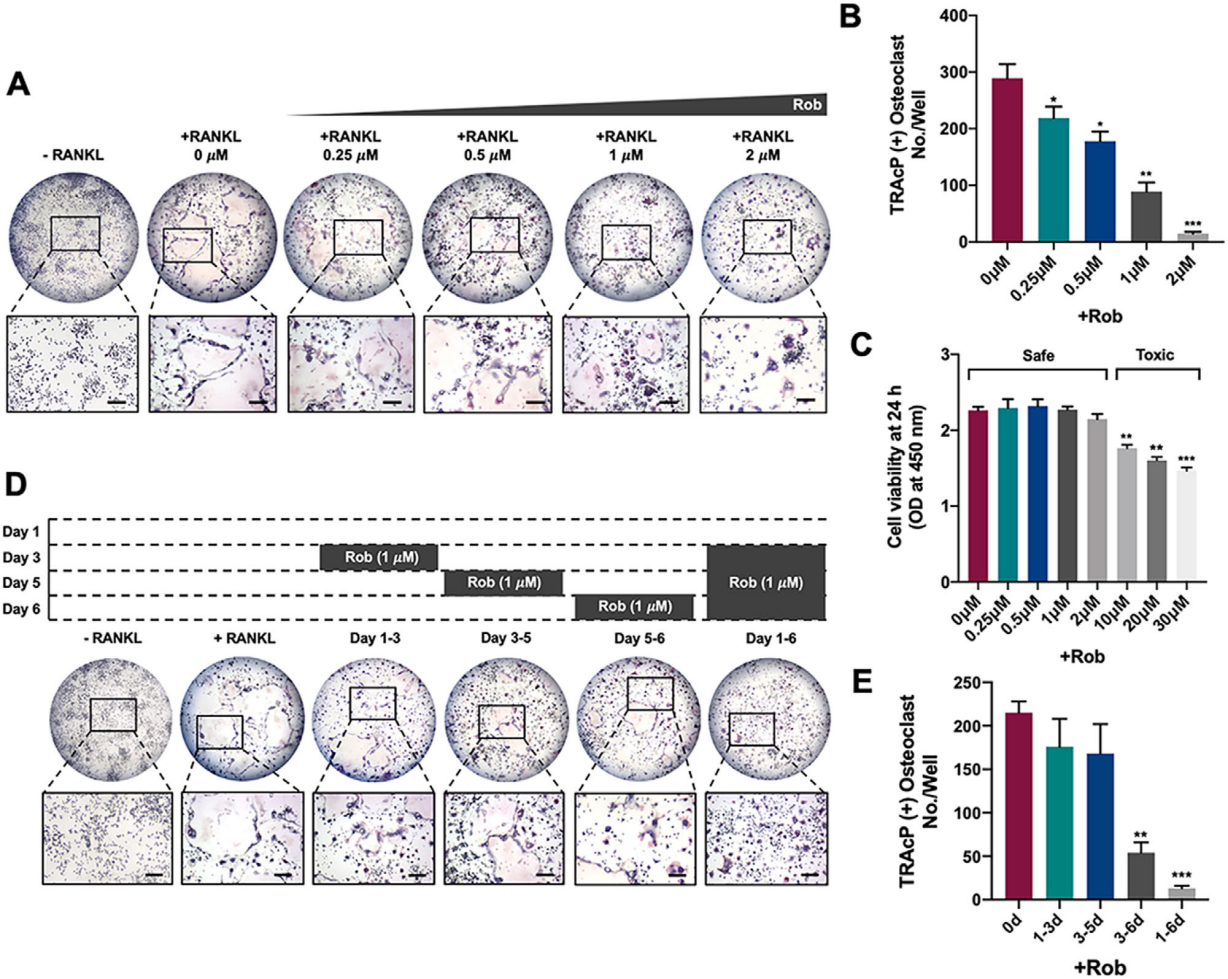
Rob attenuates RANKL‐induced osteoclast differentiation, restored podosome belt formation, and attenuated hydroxyapatite resorption. (A) Representative images showing RANKL‐induced osteoclasts treated with Rob at indicated concentrations as examined by TRAcP staining. Scale bar: 100 μM. (B) Quantitative analysis of TRAcP‐positive multinucleate (more than three nuclei) cells (*n* = 3). (C) Optical density (OD) values indicating the effects of Rob treatment for 48 h on cell viability of bone marrow macrophages (BMMs), as examined by MTS assay (*n* = 3). (D) Representative images showing BMMs treated with Rob at IC_50_ (1 μM) for the indicated periods as examined by TRAcP staining. Scale bar: 100 μM. (E) Quantitative analysis of stained multinucleate (more than three nuclei) osteoclast‐like cells treated with Rob at IC_50_ (1 μM) for the indicated periods (*n* = 3). *p < 0.05, **p < 0.01, ***p < 0.001, versus RANKL‐treated control. Abbreviation: OD, optical density

To evaluate the effects of Rob on RANKL‐induced osteoclastogenesis as well as its cytotoxicity, BMMs treated with different dosages of Rob were incubated to evaluate the inhibitory effects of Rob on osteoclasts. The amount of TRAcP+ osteoclasts increased gradually in the positive control group, but were markedly suppressed following Rob treatment in a concentration‐dependent manner (Figures 2A and 2B). The half‐maximal inhibitory concentration (IC_50_) of Rob on osteoclasts was 1μM. BMM viability was further measured after treating cells with increasing concentrations of Rob by MTS assay. As indicated in Figure 2C, Rob showed no cytotoxicity to BMMs if the concentration was lower than 10 μM. Rob was found to be toxic to the cells when its concentrations were 10–30μM. To examine the stage of osteoclast formation that was affected by Rob, Rob at IC_50_ was used to treat the RANKL‐induced cells for stated time intervals. Our findings demonstrated that Rob mainly exerted its suppressive effects from day 3 to 6 of osteoclastogenesis, rather than during the early stage (Figures 2D and 2E).

### 3.3 Rob inhibits osteoclast‐mediated bone resorption *in vitro*

**FIGURE 3:**
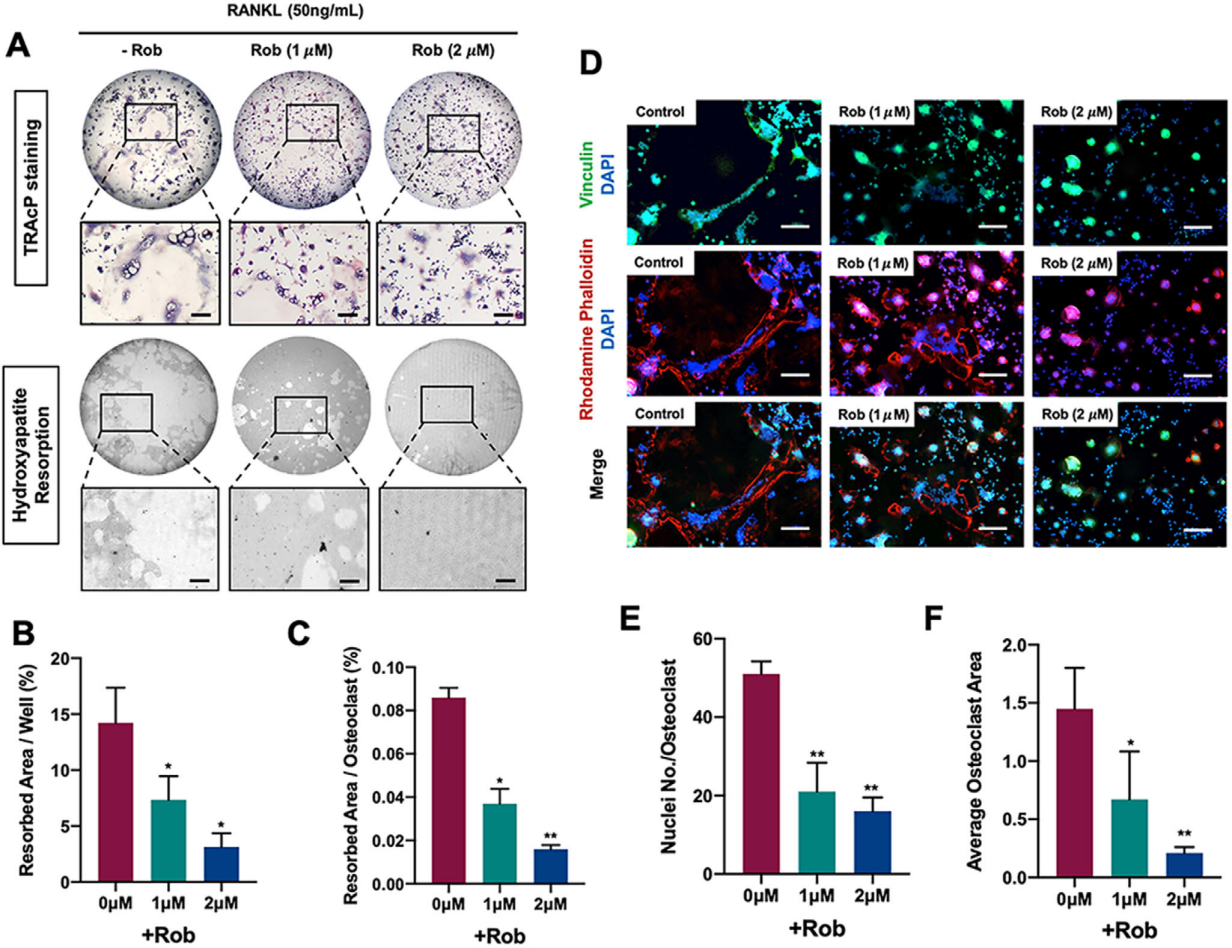
Rob restored podosome belt formation and attenuated hydroxyapatite resorption. (A) Representative images demonstrating the mature osteoclasts and hydroxyapatite bone resorption after treatment with Rob at 1 and 2 μM on hydroxyapatite‐coated plates. Scale bar: 100 μM. (B and C) Quantitative analysis of resorbed area per well in hydroxyapatite‐coated plates and resorbed area for multinucleate cells in each group (*n* = 3). (D) Representative images showing podosome belt formation after Rob addition at IC_50_ (1 μM). Vinculin (green color), F‐actin (red color), and nuclei (blue color) were stained to detect the corresponding osteoclast structures. Scale bar: 100 μM. (E) Quantitative analysis of the nuclei number per osteoclast (*n* = 3). (F) Quantitative analysis of the average osteoclast area. *p < 0.05, **p < 0.01, versus RANKL‐treated control

The effects of Rob on the resorptive function of mature osteoclasts were further examined by bone resorption assay. BMM‐derived osteoclasts were seeded with rm‐sRANKL to form multinucleated mature osteoclasts, after which equal numbers of cells were seeded onto hydroxyapatite plates. Rob significantly reduced the percentage of resorbed area per osteoclast. The number of osteoclasts was not affected by Rob at the indicated concentrations. These findings revealed that Rob inhibited osteoclast hydroxyapatite resorption, but did not cause osteoclast apoptosis (Figures 3A–2C).

### 3.4 Rob affects podosome belt formation

The formation of the podosome belt and morphological changes of RANKL‐induced osteoclasts treated with Rob were observed. It was demonstrated that the mature osteoclasts formed a clear and complete nuclear podosome belt with stimulation of rm‐sRANKL. After Rob treatment (1 or 2 μM), smaller osteoclasts with fewer nuclei were observed (Figures 3D–3F).

### 3.5 Rob causes cell apoptosis and cell cycle arrest in osteoclasts at extremely high doses

**FIGURE 4:**
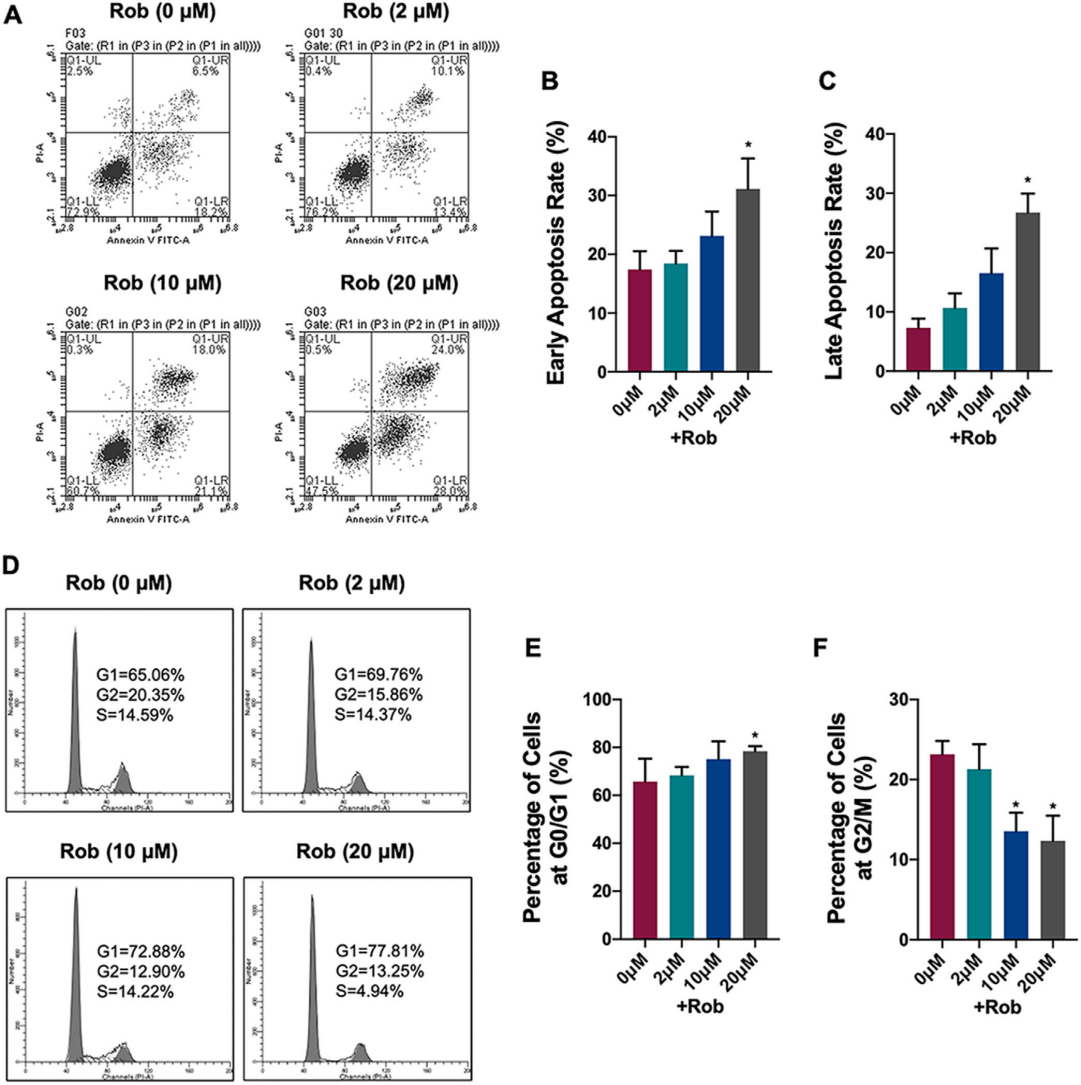
Rob induces cell apoptosis and cell cycle arrest in RANKL‐induced osteoclasts at extremely high doses. (A) Images of flow cytometry analysis of annexin‐V/PI staining in RANKL‐induced osteoclasts treated with Rob (0, 2, 10, and 20 μM). (B and C) Quantitative analysis of early and late cell apoptosis rate in RANKL‐induced osteoclasts (*n* = 3). (D) Flow cytometry analysis of cell cycle changes in RANKL‐induced osteoclasts after Rob treatment (0, 2, 10, and 20 μM). (E and F) Quantitative analysis of RANKL‐induced osteoclasts at G0/G1 and G2/M (n = 3). *p < 0.05 versus RANKL‐treated control

To further determine if Rob suppressed osteoclast differentiation via cell apoptosis, flow cytometry was employed to analyze cell apoptosis and cell cycle in RANKL‐induced osteoclasts. Our findings in FITC‐Annexin‐V/PI stain findings suggested that Rob at concentrations of 2 and 10 μM did not cause cell apoptosis, while the early and late cell apoptosis rates were both enhanced after Rob treatment at a concentration of 20 μM (Figures 4A–4C). In cell cycle changes of RANKL‐induced osteoclast, Rob at concentrations of 10 and 20 μM, but not 2 μM, markedly promoted G0/G1 cell proportion or downregulated G2/M cell proportion (Figures 4D–4F). Therefore, it was indicated that Rob induced cell apoptosis and cell cycle arrest in RANKL osteoclasts only if it admonished at extremely high concentrations. The inhibitory effect of Rob on RANKL‐induced osteoclasts at low concentrations might not come from induction of cell apoptosis.

### 3.6 Rob inhibits ROS levels by downregulating the TRAF6/Rac1/NOX1 signaling pathway and enhancing the expression of antioxidant enzymes

**FIGURE 5:**
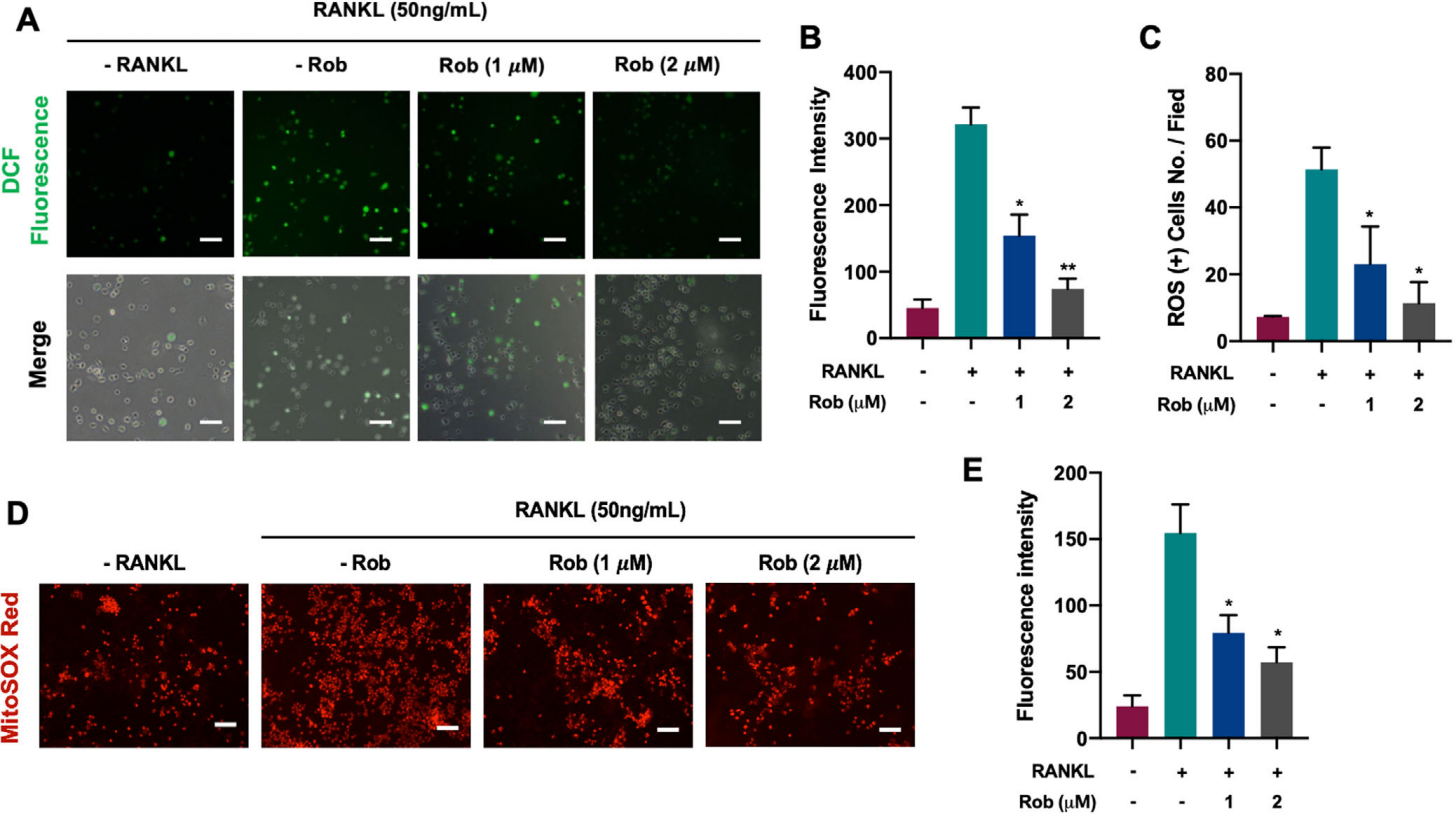
Rob reduces mitochondrial reactive oxygen species (ROS) levels in RANKL‐induced osteoclasts. (A) Representative images showing RANKL‐induced intracellular ROS generation in bone marrow macrophages (BMMs), as detected by the cell permeable oxidation‐sensitive dye H_2_DCFHDA. The upper panel shows the production of fluorescent DCF (green color). The lower panel shows the merged images of DCF fluorescence and DIC in each group. Scale bar: 100 μM. (B) Quantitative analysis of DCF fluorescence intensity per osteoclast (*n* = 3). (C) Quantitative analysis of the number of ROS stained cells in each filed (*n* = 3). (D) Representative images showing RANKL‐induced intracellular ROS generation in BMMs, as detected by the cell permeable oxidation‐sensitive dye H_2_DCFHDA. The upper panel shows the production of fluorescent DCF (green color). The lower panel shows the merged images of DCF fluorescence and DIC in each group. Scale bar: 100 μM. (E) Quantitative analysis of MitoSox Red fluorescence intensity (n = 3). *p < 0.05, **p < 0.01 versus RANKL‐treated control

We analyzed whether Rob had an effect on RANKL‐induced ROS production using a cell‐permeable H_2_DCFDA fluorescence probe. Intracellular ROS expression in the RANKL‐treated group was higher than in the control one. In contrast, the dichlorodihydrofluorescein (DCF) fluorescence intensity of each positive cell decreased after Rob treatment (Figures 5A–5C). To further determine if the origin of ROS, MitoSOX Red reagent was used to specifically detect mitochondria in live cells. Osteoclasts pretreated with Rob at 5 and 10 μM displayed decreased MitoSOX Red fluorescence upon RANKL stimuli, indicating lower levels of mitochondrial superoxide production (Figures 5D–5E).

**FIGURE 6:**
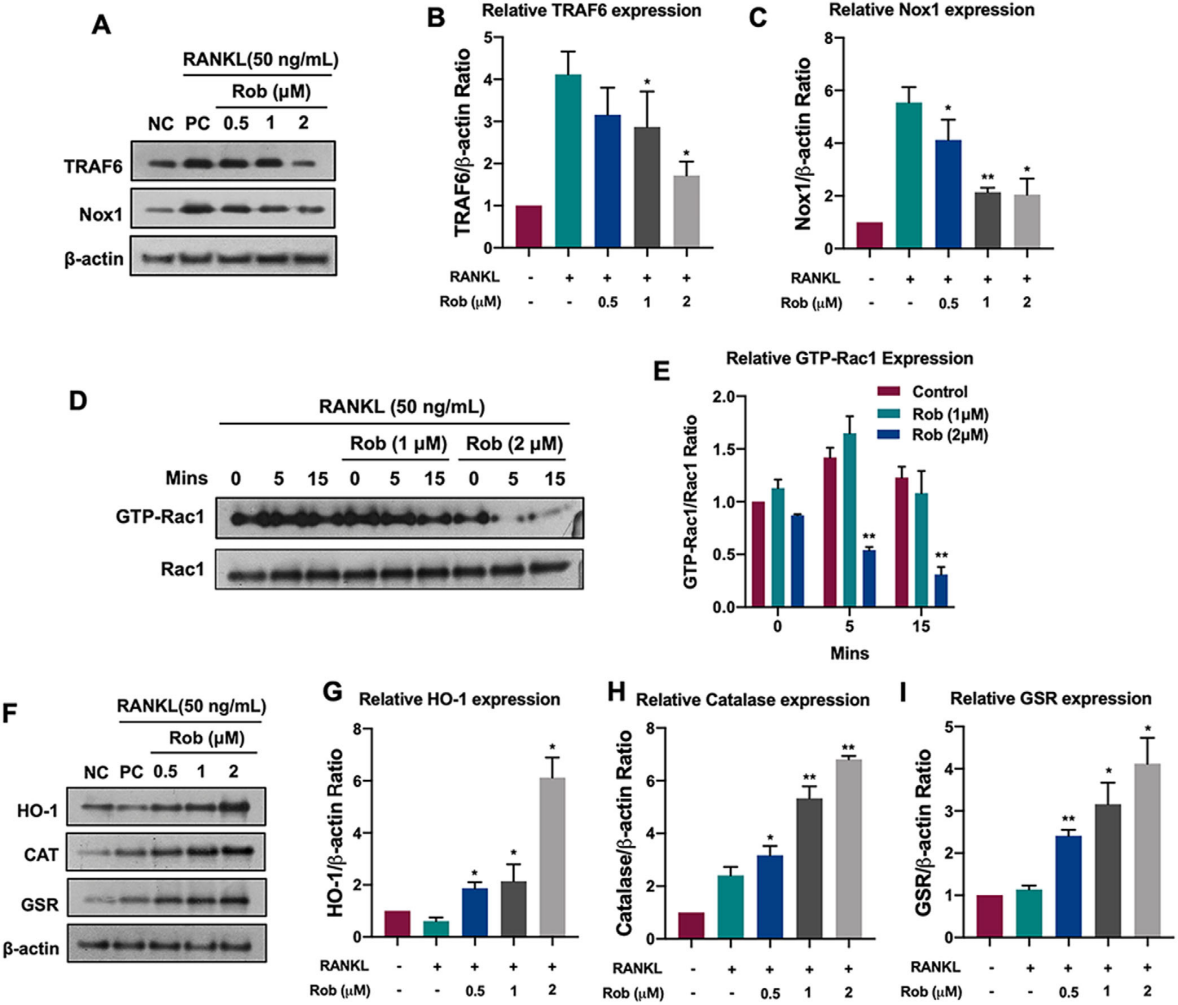
Rob regulates intracellular reactive oxygen species (ROS) signaling in RANKL‐induced osteoclasts. (A) Representative images of TRAF6 and NOX1 expression in RANKL‐treated osteoclasts after the addition of Rob (0.5, 1, and 2 μM). Protein expression is normalized to β‐actin. (B and C) Quantitative analysis of TRAF6 and NOX1 normalized to β‐actin (*n* = 3). (D) Representative images of GTP‐Rac1 expression at different time points (0, 5, and 15 min) after Rob treatment (1 and 2 μM). Cells were cultured with PAK1‐PBD protein. Protein expression is normalized to Rac1 levels. (E) Quantitative analysis of GTP‐Rac1 normalized to Rac1 (*n* = 3). (F) Representative images of HO‐1, CAT, and GSR. (G–I) Quantitative analysis of protein band signal intensity of HO‐1, CAT, and GSR normalized to β‐actin (*n* = 3). *p < 0.05, **p < 0.01 versus RANKL‐treated control Abbreviations: CAT, catalase; GSR, glutathione‐disulfide reductase; GTP, guanosine‐5′‐triphosphate; HO‐1, heme oxygenase‐1; NOX, nicotinamide adenine dinucleotide phosphate oxidase; Rac1, Ras‐related C3 botulinum toxin substrate 1; TRAF6, TNF receptor‐associated factor 6.

In order to investigate the mechanism of ROS regulation by Rob, we studied the activation level of NOX1, which was the main ROS‐producing factor. Our findings demonstrated that RANKL markedly upregulated NOX1 expression, whereas Rob inhibited this upregulation (0.5, 1, and 2 μM) (Figures 6A and 4C). Because TRAF6 and GTP‐bound Rac1 (GTP‐Rac1) were necessary to activate NOX1, we further evaluated whether Rob could inhibit NOX1 activity by inhibiting TRAF6 and GTP‐Rac1 activity. RANKL increased TRAF6 expression, while Rob (0.5, 1, and 2 μM) attenuated it (Figures 6D–6E). GTP‐Rac1, the cytoplasmic component of NOX1, is responsible for the activation of NOX1. Our results revealed that GTP‐Rac1 activation increased immediately after 5 min of rm‐sRANKL stimulation, but slightly decreased after 15 min. This activation was greatly inhibited after Rob treatment in a dose‐dependent manner (1 and 2 μM) (Figures 6D and 6E). Finally, in order to verify whether Rob could reduce ROS levels by upregulating antioxidant enzymes, we measured the expression levels of several enzymes, like HO‐1, catalase (CAT), and glutathione‐disulfide reductase (GSR). The results indicated that RANKL stimulation partially reduced the expression of these enzymes, which were restored in RANKL‐induced osteoclasts after Rob treatment (Figures 6F–6I). Collectedly, our data revealed that Rob reduced RANKL‐induced intracellular ROS levels in two ways: suppression of ROS production and enhancement of ROS scavenging.

### 3.7 Rob attenuates NF‐κB, MAPK, and NFATc1 activity as well as downstream protein expression

**FIGURE 7:**
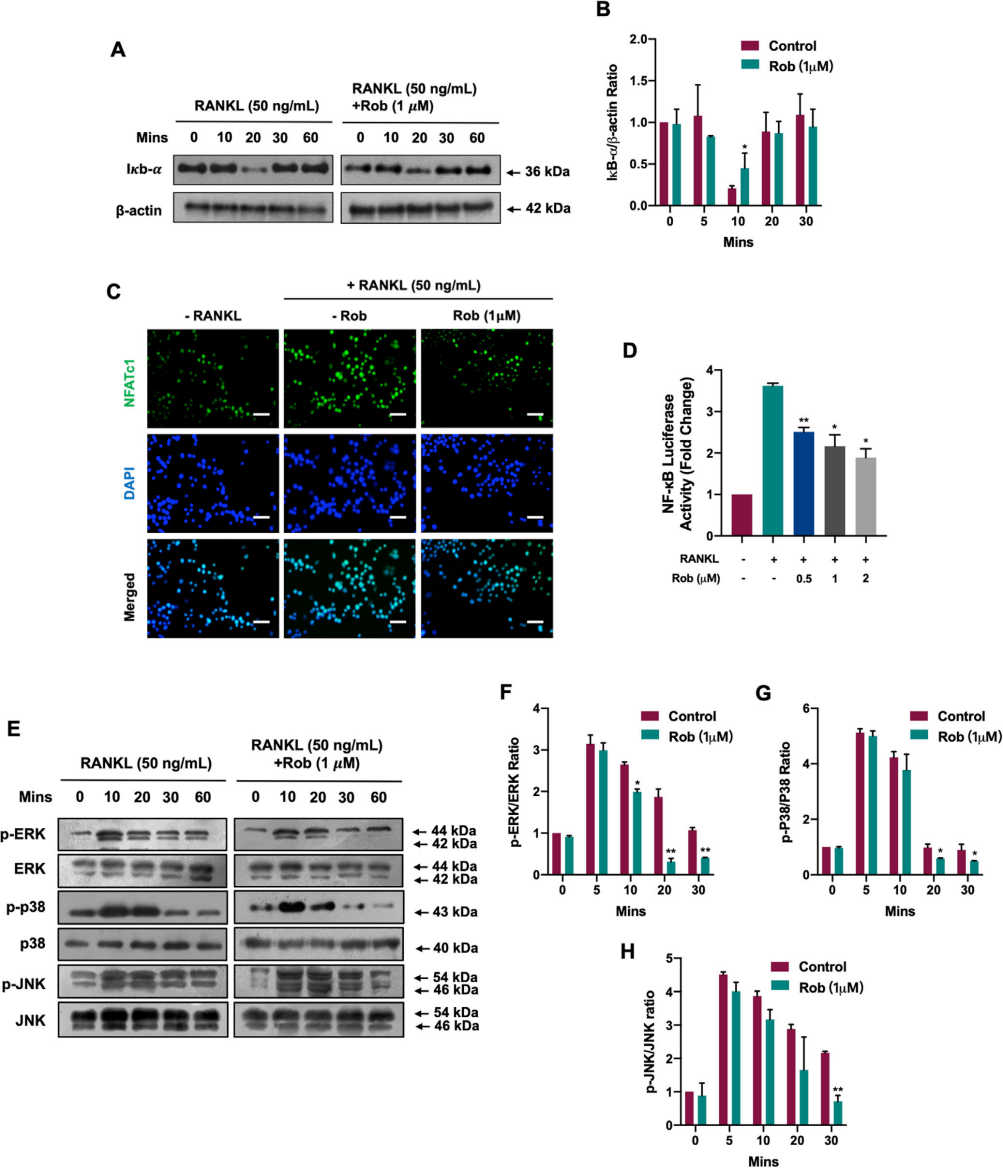
Rob suppresses the activation of NF‐κB and MAPK signaling during osteoclast differentiation. (A) Representative images of RANKL‐mediated IκBα degradation in bone marrow macrophages (BMMs) treated with Rob at IC_50_ (1 μM). (B) Quantitative analysis of IκBα expression normalized to β‐actin (*n* = 3). (C) Immunofluorescence staining showing the Rob (1 μM) affected the RANKL‐induced nuclear translocation of p65. Scale bar: 100 μM. (D) NF‐κB luciferase assay demonstrating Rob (0.25, 0.5, 1, and 2 μM) regulated RANKL‐mediated NF‐κB transcription (*n* = 3). (E) Representative images of phosphorylation of ERK, P38, and JNK, which are involved in MAPK signaling. BMMs were pretreated with Rob at IC_50_ (1 μM) prior to the stimulation by RANKL, and after 10, 20, 30, and 60 min, the total protein was analyzed by western blot. (F–H) Quantitative analysis of phosphorylation of ERK, p38, and JNK normalized to total ERK, p38, and JNK (*n* = 3). *p < 0.05, **p < 0.01 versus RANKL‐treated control

NFATc1 activity depends on NF‐κB signal transduction and is regulated by the MAPK pathway. In order to explore Rob's effects on the NF‐κB pathway when stimulated by RANKL, we measured the protein levels of IκB‐α, an inhibitor of NF‐κB. Rob was found to delay the RANKL‐stimulated degradation of IκB‐α (Figures 7A and 7B). By using immunofluorescence staining, it was found that more p65 translocation into the nucleus than in the cytoplasm was markedly decreased by Rob (1 μM), which was supported by the luciferase gene assay of NF‐κB activity. The findings indicated that Rob had a positive impact on the p65 nuclear translocation of IκB‐α (Figures 7C and 7D). We also examined RANKL's inhibition of the MAPK signaling pathway during osteoclast differentiation, and found that Rob significantly reduced the proportions of phosphorylated ERK, p38, and JNK with respect to their total protein levels, respectively (Figures 7E–7H). These data showed that Rob inhibited RANKL‐stimulated NF‐κB activity and MAPK phosphorylation.

**FIGURE 8:**
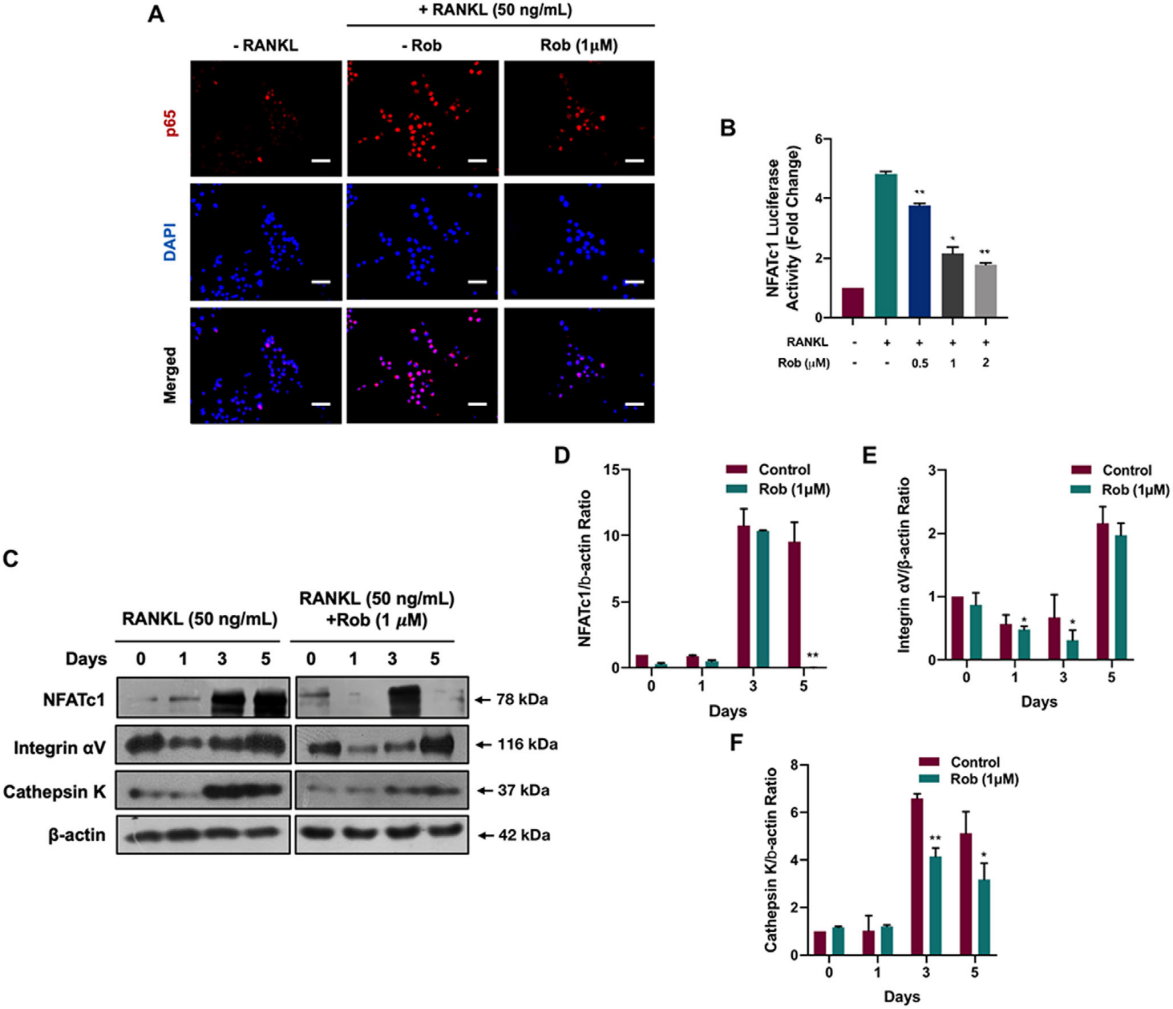
Rob suppresses the activation of NFATc1 signaling during osteoclast differentiation. (A) Immunofluorescence staining demonstrating the Rob (1 μM) modulated the RANKL‐induced nuclear translocation of NFATc1. Scale bar: 100 μM. (B) NFATc1 luciferase assay showing that Rob (0.5, 1, and 2 μM) affected the RANKL‐induced nuclear translocation of NFATc1 (*n* = 3). (C) Representative images of NFATc1 and the downstream proteins Integrin αV, and Cathepsin K. Bone marrow macrophages (BMMs) were pretreated with Rob at IC_50_ (1 μM) prior to the stimulation with RANKL. Total protein was analyzed by western blot at 1, 3, and 5 days. (D–F) Quantitative analysis of protein expression levels of NFATc1, Integrin αV, and Cathepsin K normalized to β‐actin (*n* = 3). *p < 0.05, **p < 0.01 versus RANKL‐treated control

In order to investigate the mechanisms by which Rob suppressed osteoclastogenesis, we measured NFATc1 activity by a immunofluorescence staining and a luciferase assay. It was indicated that NFATc1 was less nucleus translocation in Rob‐treating group (Figure 8A). RAW 264.7 transfected with an NFATc1 gene reporter were incubated with various concentrations of Rob. The results suggested that Rob (0.5, 1, and 2 μM) greatly blocked the nuclear translocation of NFATc1 (Figure 8A). In addition, Rob significantly decreased the protein levels of NFATc1, which was upregulated after 3 and 5 days of RANKL treatment (Figures 8C and 7D). In addition, the expressions of downstream factors related to osteoclastogenesis, such as Cathepsin K and Integrin αV, were simultaneously downregulated by Rob at 3 and 5 days (Figures 7C–7F).

### 3.8 Rob reduces Ca^2+^ oscillations and suppresses expression of osteoclast‐specific genes

**FIGURE 9:**
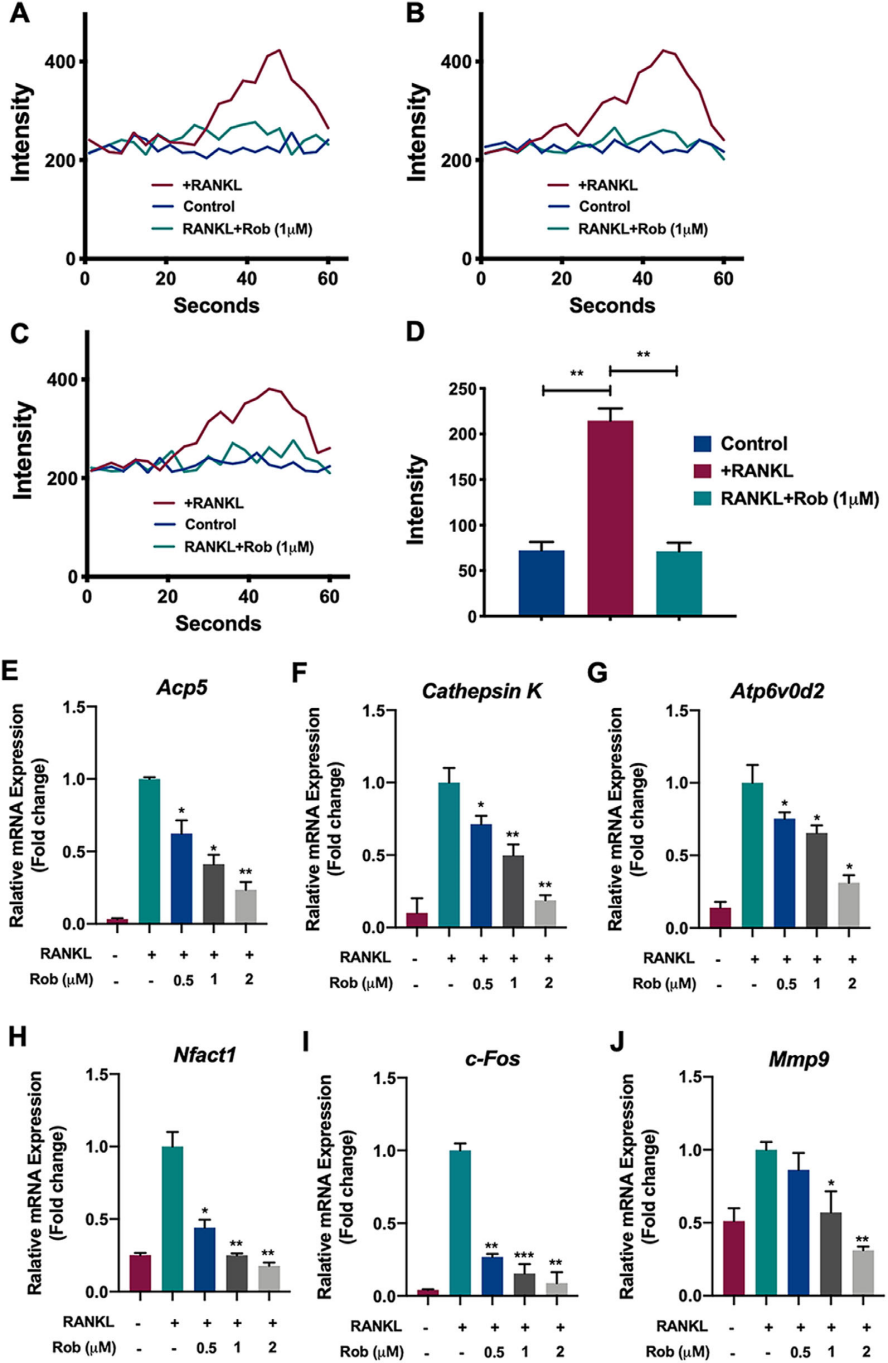
Rob inhibits RANKL‐induced Ca^2+^ oscillation and regulated specific osteoclast‐related gene expression. (A–C) Calcium flux in bone marrow macrophages (BMMs) stimulated by RANKL and Rob at IC_50_ (1 μM) was measured with the Fluo4 calcium indicator. Representative images showing temporal fluorescence profiles of single cells in three repeated experiments  . (D) Quantitative analysis of average changes of fluorescence intensity (maximum value–minimum value) in oscillating cells (*n* = 3). **(E–J)** RT‐qPCR was performed to identify the expression of osteoclast‐specific genes in RANKL‐induced osteoclasts, including *Acp5* (E), *Cathepsin K* (F), *Atp6v0d2* (G), *Nfact1* (H), *c‐Fos* (I), and *Mmp9* (J). Gene expression was normalized to *GAPDH* (*n* = 3). *p < 0.05, **p < 0.01, ***p < 0.001 versus RANKL‐treated control

Increasing Ca^2+^ levels induced by RANKL contributed to NFATc1 activation. Because Rob was found to attenuate expression of NFATc1 in osteoclast, we further investigated the potential effects of Rob on cytoplasmic Ca^2+^ oscillations. As expected, the enhancement of RANKL‐mediated Ca^2+^ oscillations was reduced by nearly 50%–60% after treatment with Rob (1 μM), which was consistent with its inhibition of NFATc1 activation (Figures 9A–9D).

Quantitative PCR analysis showed that during osteoclast differentiation of BMMs, mRNA levels of the osteoclast‐related genes Acp5, *Cathepsin K*, Atp6v0d2, *Nfact1*, *c‐Fos*, and *Mmp9* were all enhanced. After Rob treatment, the expression of these genes during RANKL‐induced osteoclastogenesis was inhibited (Figures 9E–9J). Our findings revealed that Rob inhibited the activation of downstream osteoclast‐related genes, thus inhibiting the development and proliferation of osteoclasts *in vitro*.

### 3.9 Rob prevents ovariectomy‐induced bone loss

In addition, we identified the therapeutic effects of Rob on bone loss using OVX osteoporotic mice treated with 6 mg/kg Rob every 2 days. No serious adverse events or mortality were observed in the mice during the OVX procedure or the period of intraperitoneal Rob injection. Moreover, Rob caused no side effects on mice's body weight (Figure S4), laboratory biochemistry (Table S3), or hemograms (Table S4). Lungs, livers, kidneys, hearts, and spleens from the mice in each group were isolated, and no differences in the size or surface gloss were observed between the groups (Figures S5 and S6).

**FIGURE 10:**
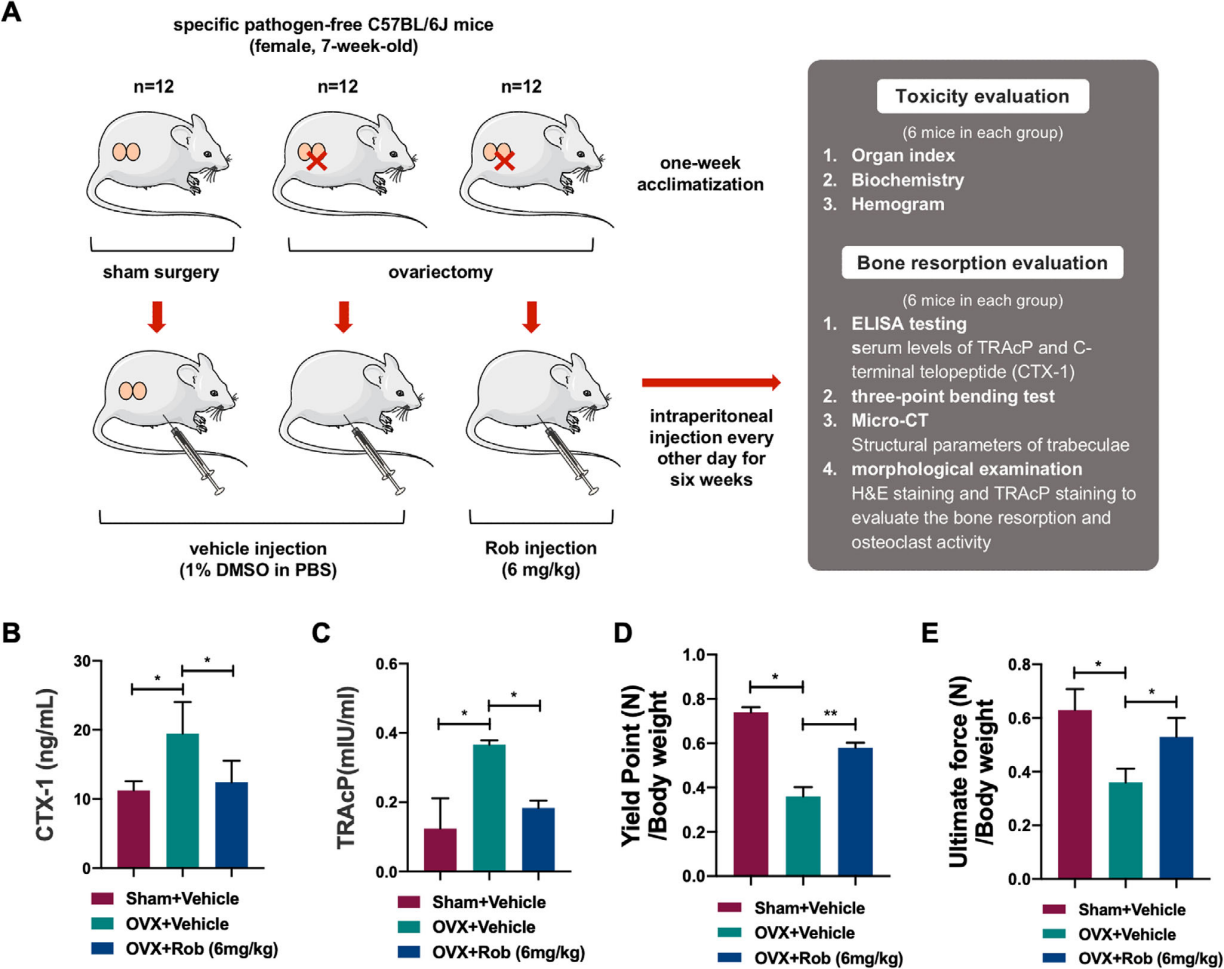
Injection of Rob prevents bone loss in OVX model. (A) Flowchart demonstrating the *in vivo* study design to identify Rob's therapeutic effects. **(B and C)** Quantitative analysis of the serum levels of tartrate‐resistant acid phosphatase (TRAcP) and C‐terminal telopeptide (CTX‐1) (*n* = 6). (D and E) Quantitative analysis of the ultimate force (*N*) and yield point (*N*) by body weight of mice (*n* = 6). *p < 0.05 and **p < 0.01 relative to controls and OVX‐untreated controls Abbreviations: BV/TV, bone volume per tissue volume; Conn.Dn, connectivity density; Tb. N, number of trabeculae; Tb.Th, trabecular thickness.

**FIGURE 11:**
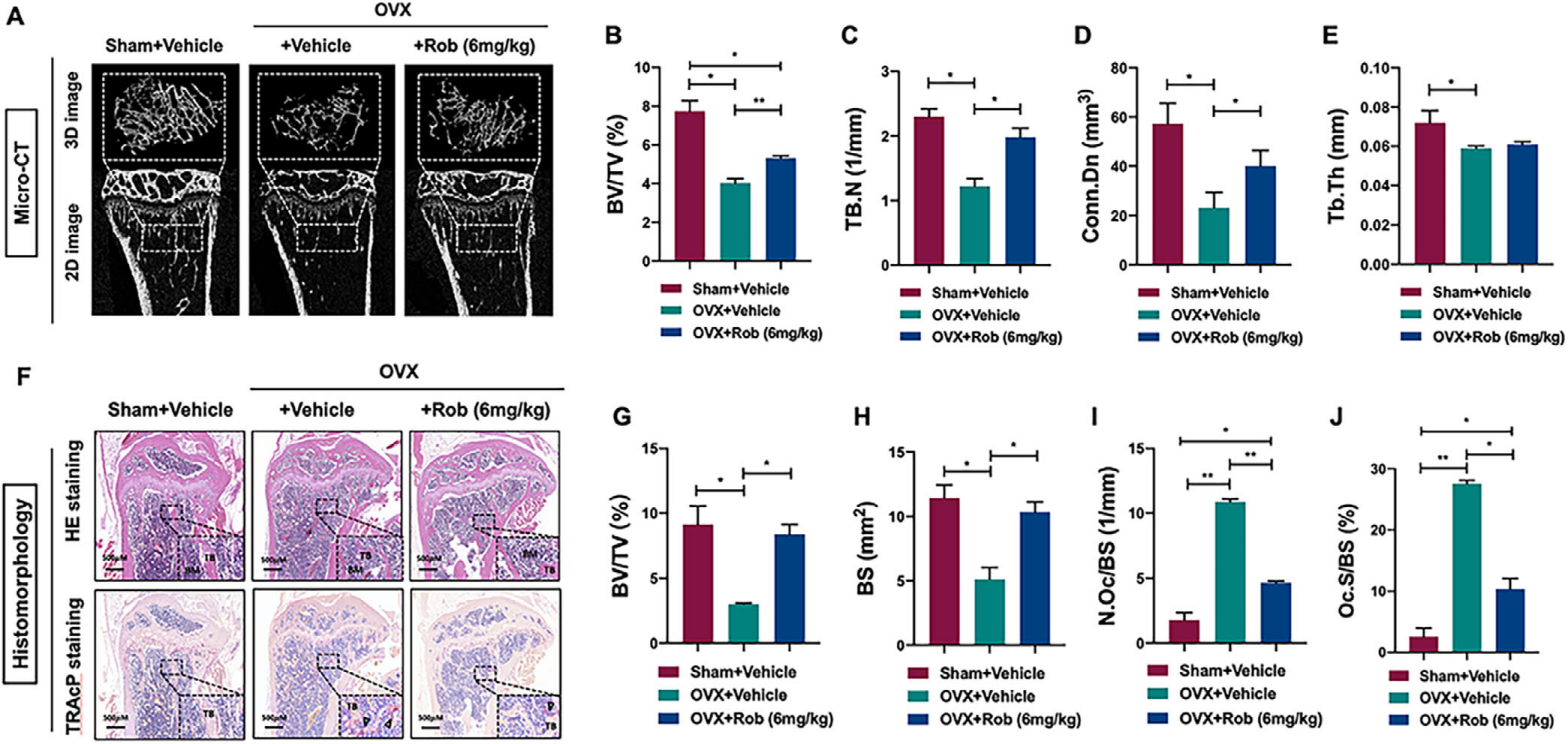
Injection of Rob prevents bone loss in OVX model. (A) Representative micro‐CT images indicating that bone loss was rescued by intraperitoneal Rob injection. (B–E) Quantitative analyses of bone structural parameters (BV/TV, Tb.N, Conn.Dn, and Tb.Th) (*n* = 6). (F) Representative images of H&E and TRAcP staining of bone slices. (G–J) Quantitative analyses of BV/TV, BS, N.Oc/BS, and Oc.S/B (*n* = 6). *p < 0.05 and **p < 0.01 relative to controls and OVX‐untreated controls Abbreviations: N.Oc/BS, number of osteoclasts /bone surface; Oc.S/BS, osteoclast surface/bone surface.

Compared with the sham‐operated group, serum TRAcP and CTX‐1 were reduced by Rob in the OVX mice model (Figures 10B and 10C). The OVX mice showed significant bone loss in the tibia, a sharp decrease in the bone volume/total volume ratio, and deterioration of the trabecular bone structure; that is, a decrease in trabecular number and an increase in connective density were identified. A three‐point bending test in the tibias of each mice group demonstrated that the enhancement of the yield point (the mechanical force leading to complete destruction to the bone matrix) as well as the ultimate force (indicating the integrity of the bone) (Figures 10D and 10E). Treatment of Rob reversed the decrease in bone matrix and prevented the estrogen deficiency induced bone loss *in vivo* (Figures 11A–11E). In order to evaluate the change in osteoclast formation in the mice after Rob treatment, we performed TRAcP staining on the bone slices of isolated tibia. The findings demonstrated that Rob suppressed the enhancement of the osteoclast surface/bone surface ratio, as well as the number of osteoclasts/bone surface area ratio caused by the OVX procedure, indicating that Rob could reduce the activity of osteoclasts *in vivo* (Figures 11F–11J). These results demonstrated that Rob rescued estrogen deficiency‐induced bone resorption by suppressing osteoclast activity.

**FIGURE 12:**
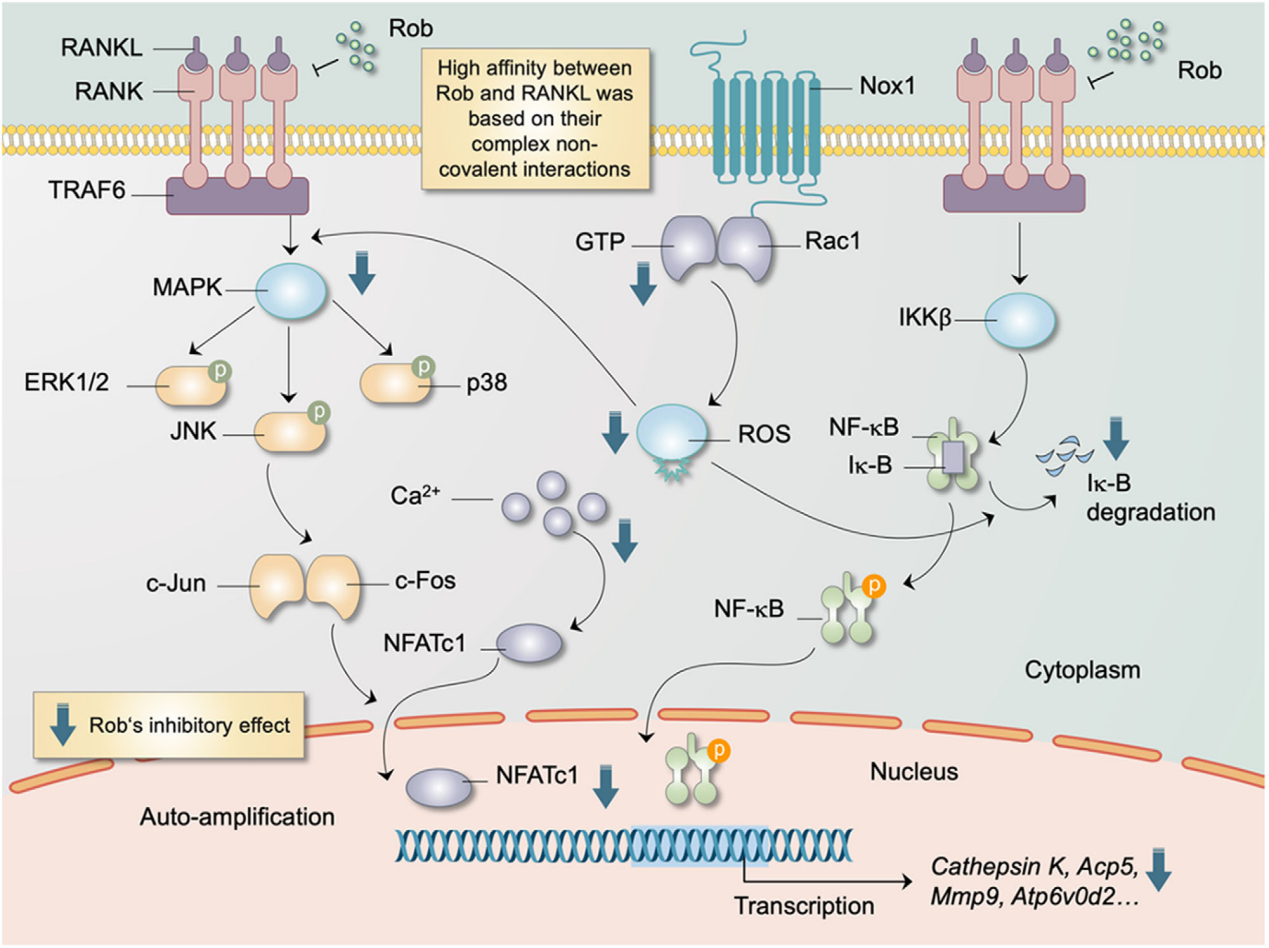
Schematic diagram for the molecular regulation of Rob in RANKL‐induced osteoclastogenesis. Upon Rob's blockage of the combination of RANKL and RANK, MAPK and NF‐κB pathways as well as calcium oscillation are suppressed, resulting in attenuation of nucleus translocation and auto‐amplification of NFATc1. Generation of reactive oxygen species (ROS) is inhibited while the scavenging of ROS is enhanced by antioxidant enzymes, both of which are regulated by Rob Abbreviations: ERK, extracellular signal‐regulated kinase; GTP, guanosine‐5′‐triphosphate; JNK, c‐Jun N‐terminal kinase; MAPK, mitogen‐activated protein kinase; NFATc1, nuclear factor of activated T cells 1; NOX, nicotinamide adenine dinucleotide phosphate oxidase; Rac1, Ras‐related C3 botulinum toxin substrate 1; TRAF6, TNF receptor‐associated factor 6.

## 4 DISCUSSION

Excessive osteoclast formation and enhanced bone loss are the primary contributors to osteoporosis incidence.^3^ Thus far, various studies have identified therapeutic drugs to prevent osteoclast‐mediated bone resorption, such as hormone‐like medications,^22^ bisphosphonates,^23^ denosumab,^24^ and teriparatide,^25^ but many adverse effects have been reported. Therefore, it is very urgent to explore alternative natural medicines to improve osteolysis therapy. In the present study, we found that the natural compound Rob, interacting with RANKL, prevented OVX‐induced bone destruction by suppressing osteoclast activity via the classical RANKL‐mediated signaling pathways, including the ROS, NF‐κB, MAPKs, and NFAcT1 signaling pathways, both *in vitro* and *in vivo* (Figure 12).

After RANKL stimulation, Rob treatment can significantly reduce (i) cellular ROS levels, and (ii) the activity of NF‐κB and MAPKs, leading to a reduction in NFATc1 activity. The intracellular ROS levels are determined by ROS generation and scavenging.^26^ ROS are produced during the osteoclastogenesis procedure via downstream signaling, involving factors such as NOX1, TRAF6, and Rac1.^11^ NOX1‐mediated ROS generation regulates RANKL‐induced signal transduction, which is necessary for the osteoclast activity.^11^, ^27^ Therefore, we speculate that the downregulation of ROS levels may partly depend on the inhibition of NOX1 expression. Our results revealed that Rob effectively reduced the expression of NOX1 by decreasing GTP‐Rac1 levels. Furthermore, a variety of antioxidant enzymes have been shown to reduce oxidative stress in osteoclastogenesis and to play critical roles in the maintenance of redox balance. For example, the induction of HO‐1 induced by oxidants and mediators of inflammation has been postulated to be a suppressive mechanism of osteoclastogenesis.^28^ The NADPH‐dependent GSR catalytically converts glutathione disulfide back into glutathione, the downregulation of which is found to activate the NF‐κB transcription factor.^29^ Moreover, enhancement of GSR activity is indicated to be stimulated by estradiol in osteoclasts; this process is mediated by ERKs.^30^ CAT, which is regulated by FOXO (a subclass of the Forkhead transcription factors), greatly contributes to the detoxification of hydrogen peroxide and subsequently blocks RANKL‐induced ROS generation.^31^ In the present study, the expression of these ROS scavengers in RANKL‐induced cells was upregulated by Rob. Nevertheless, the mechanisms by which antioxidant enzymes were upregulated remain unknown. In summary, Rob suppressed ROS levels in osteoclasts by restraining ROS generation and promoting ROS scavenging.

An increasing body of evidence shows that RANKL increases ROS levels, regulating the signal cascade of the MAPK and NF‐κB signaling pathway.^10^, ^11^, ^32^, ^33^, ^34^ NF‐κB mainly contributes to early osteoclast development and is followed by the activation of c‐Fos and NFATc1.^35^, ^36^ The absence of NF‐κB signal transduction leads to failure of osteoclastogenesis and an osteogenic phenotype in mice.^37^, ^38^ TNFα‐induced NF‐κB activation is regulated by redox‐dependent regulation of dynein light chain LC8. ROS can oxidize LC8 into a dimer, which is connected by a disulfide bond between Cys‐2 residues on each subunit, promoting its dissociation from IκBα, and thus allowing IκB kinase degradation, which in turn causes release of NF‐κB dimer and allows NF‐κB/NFATc1 transfer into the nucleus.^33^ In the present study, Rob treatment effectively inhibited the RANKL‐induced degradation of IκB‐α. The high IκB‐α expression in the Rob treatment groups indicates that IκB‐dependent NF‐κB inactivation plays a critical role in the inhibition of osteoclast formation by Rob. In addition, the luciferase assay results convincingly show that Rob indeed inhibits the activation of NF‐κB transcription.

MAPK proteins, including ERK, p38, and JNK, are proline‐mediated serines.^39^, ^40^ They are reported to be key molecules in intracellular signaling pathways and regulate osteoclastogenesis functions such as cell growth, development, and differentiation.^41^, ^42^ Stimulation of the ERK signal promotes the transcription of *c‐Fos*, thus prolonging osteoclast survival and preventing apoptosis.^43^ Both phosphorylated JNK and p38 could induce the differentiation, fusion, and activation of osteoclasts. In contrast, inhibition of JNK and p38 phosphorylation blocks osteoclast formation as well as RANKL‐induced bone resorption.^44^, ^45^, ^46^, ^47^ In addition, ROS, triggered by RANKL as a second bio‐messenger, is speculated to oxidatively modify MAPK signaling and degrade MAPK phosphatases (MKPs).^48^ Our findings illustrated that Rob suppressed the phosphorylation of MAPK involved in the MAPK signaling pathway, suggesting that Rob suppressed NFATc1 expression through MAPK signaling, thus downregulating the expression of Integrin αV, Cathepsin K, and other functional proteins.

NFATc1, a well‐known transcription factor in osteoclast differentiation and proliferation, is considered to be a key RANKL‐induced signal transducer.^49^, ^50^ In addition, NFATc1's regulation of the expression of osteoclast markers driven by multiple specific promoters, such as *Acp5*, *Cathepsin K*, *Atp6v0d2*, *Nfact1*, *c‐Fos*, and *Mmp9*, which were essential for osteoclast formation, was repressed by Rob.^51^, ^52^, ^53^, ^54^ In our study, it was demonstrated that the activity and nuclear translocation of NFATc1 stimulated by RANKL were significantly attenuated by Rob. Ca^2+^ levels are related to the protein synthesis of gelsolin, which is a Ca^2+^‐dependent actin binding factor that is crucial for osteoclast function.^55^ Consistently, our findings suggested that Rob inhibited the increase of RANKL‐induced Ca^2+^ oscillations, resulting in inhibition of NFATc1 activation and of self‐amplification. Therefore, we speculate that the inhibition of NFAT trans‐activation after Rob treatment partly depends on the decrease in RANKL‐induced Ca^2+^ oscillations, emphasizing the effects of Rob on osteoclast formation and differentiation.

Considering the potential effects of Rob *in vitro*, we further studied its effects in an osteoporosis model in OVX mice. OVX mice are widely applied in studies of bone resorption caused by estrogen deficiency, as OVX closely simulates the characteristics of bone changes related to postmenopausal osteoporosis. Micro‐CT and histological analysis revealed that intraperitoneal Rob injection could block the effects of estrogen deficiency by increasing bone volume and restoring the bone trabecular microstructure. Morphometric evaluation shows consistent outcomes with our *in vitro* data, that is, Rob treatment downregulates the number of TRAP‐positive osteoclasts in bone. A three‐point bending test also indicated that Rob rescues biomechanical properties of tibias in the mouse model. In addition, serum levels of TRAcP and CTX‐1, regulators that contribute to osteoclast‐induced bone loss, were downregulated *in vivo* in the Rob treatment group. Our results indicate that Rob prevents bone destruction in the mouse model due to its inhibitory effect on excessive osteoclastogenesis.

Taken together, our findings demonstrat that Robcan prevent the osteolysis in OVX animal model. Rob markedly inhibits RANKL‐mediated osteoclast production and bone resorptive function by (i) inhibiting ROS generation and (ii) NFATc1, NF‐κB, and MAPK signaling. Our findings indicate that Rob is an available therapeutic agent for osteoclast‐mediated osteoporosis.

## CONFLICT OF INTEREST

The authors declare that there is no conflict of interest that could be perceived as prejudicing the impartiality of the research reported.

## Supporting information


**FIGURE S1**. The chemical structure and formula of Rob inferred from PubChem (https://pubchem.ncbi.nlm.nih.gov).

**FIGURE S2**. Ramachandran plot images showing the status of amino acid residues before **(A)** and after **(B)** optimization of RANKL protein structure. The stability of amino acid is demonstrated as high (red), medium (yellow), and low (white).

**FIGURE S3. (A)** Representative images of H&E staining of decalcified bone sections. Mice were treated by various doses of Rob (1 mg/kg, 3 mg/kg, and 6 mg/kg) to identify the most effective concentration for bone loss prevention. **(B and C)** Quantitative analyses of BV/TV and BS in tissue sections (*n* = 6 per group).

**FIGURE S4**. Quantitative analysis of body‐weight changes of mice in each group during Rob treatment.

**FIGURE S5**. Representative images of organs of mice in each group, including lung (A), liver (B), kidney (C), heart (D), and spleen (E). Circles one to three indicate the organs of sham group, OVX group, and OVX+Rob group, respectively.

**FIGURE S6**. Histomorphology analysis of organs of mice in each group, including lung, liver, kidney, heart, and spleen.

Appendices
