# Supplementary figures and images for: A novel RANKL‐targeted flavonoid glycoside prevents osteoporosis through inhibiting NFATc1 and reactive oxygen species
Source: Clin Transl Med. 2021 May 21;11(5):e392. doi: 10.1002/ctm2.392 (PMC8140192; doi:10.1002/ctm2.392)

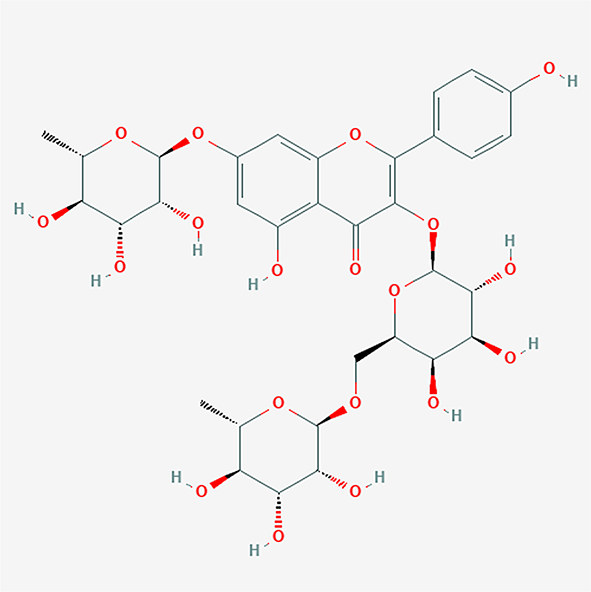

Supplement: Supplementary file 1 — FIGURE S1. The chemical structure and formula of Rob inferred from PubChem (https://pubchem.ncbi.nlm.nih.gov). [file CTM2-11-e392-s004.tif]

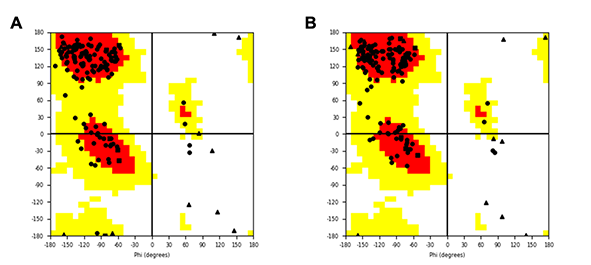

Supplement: Supplementary file 2 — FIGURE S2. Ramachandran plot images showing the status of amino acid residues before (A) and after (B) optimization of RANKL protein structure. The stability of amino acid is demonstrated as high (red), medium (yellow), and low (white). [file CTM2-11-e392-s001.tif]

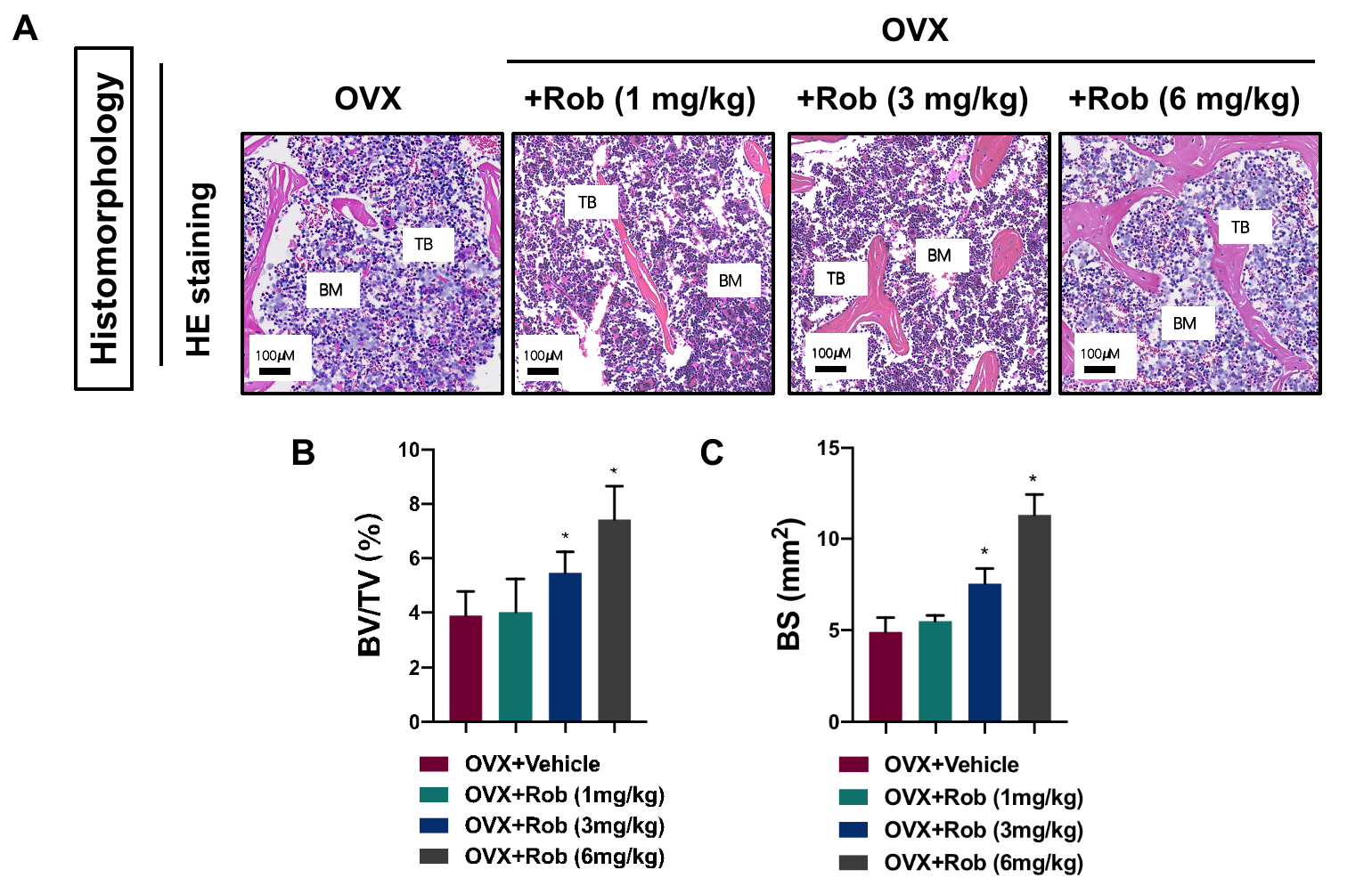

Supplement: Supplementary file 3 — FIGURE S3. (A) Representative images of H&E staining of decalcified bone sections. Mice were treated by various doses of Rob (1 mg/kg, 3 mg/kg, and 6 mg/kg) to identify the most effective concentration for bone loss prevention. (B and C) Quantitative analyses of BV/TV and BS in tissue sections (n = 6 per group). [file CTM2-11-e392-s002.tif]

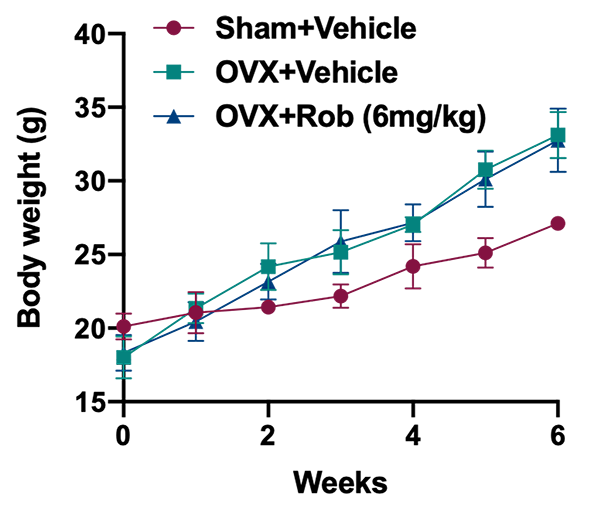

Supplement: Supplementary file 4 — FIGURE S4. Quantitative analysis of body‐weight changes of mice in each group during Rob treatment. [file CTM2-11-e392-s003.tif]

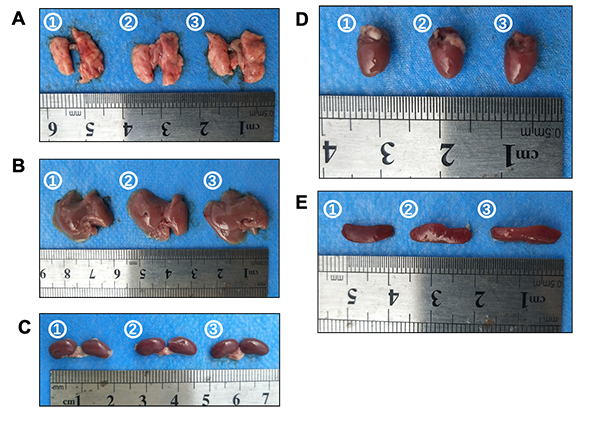

Supplement: Supplementary file 5 — FIGURE S5. Representative images of organs of mice in each group, including lung (A), liver (B), kidney (C), heart (D), and spleen (E). Circles one to three indicate the organs of sham group, OVX group, and OVX+Rob group, respectively. [file CTM2-11-e392-s005.tif]

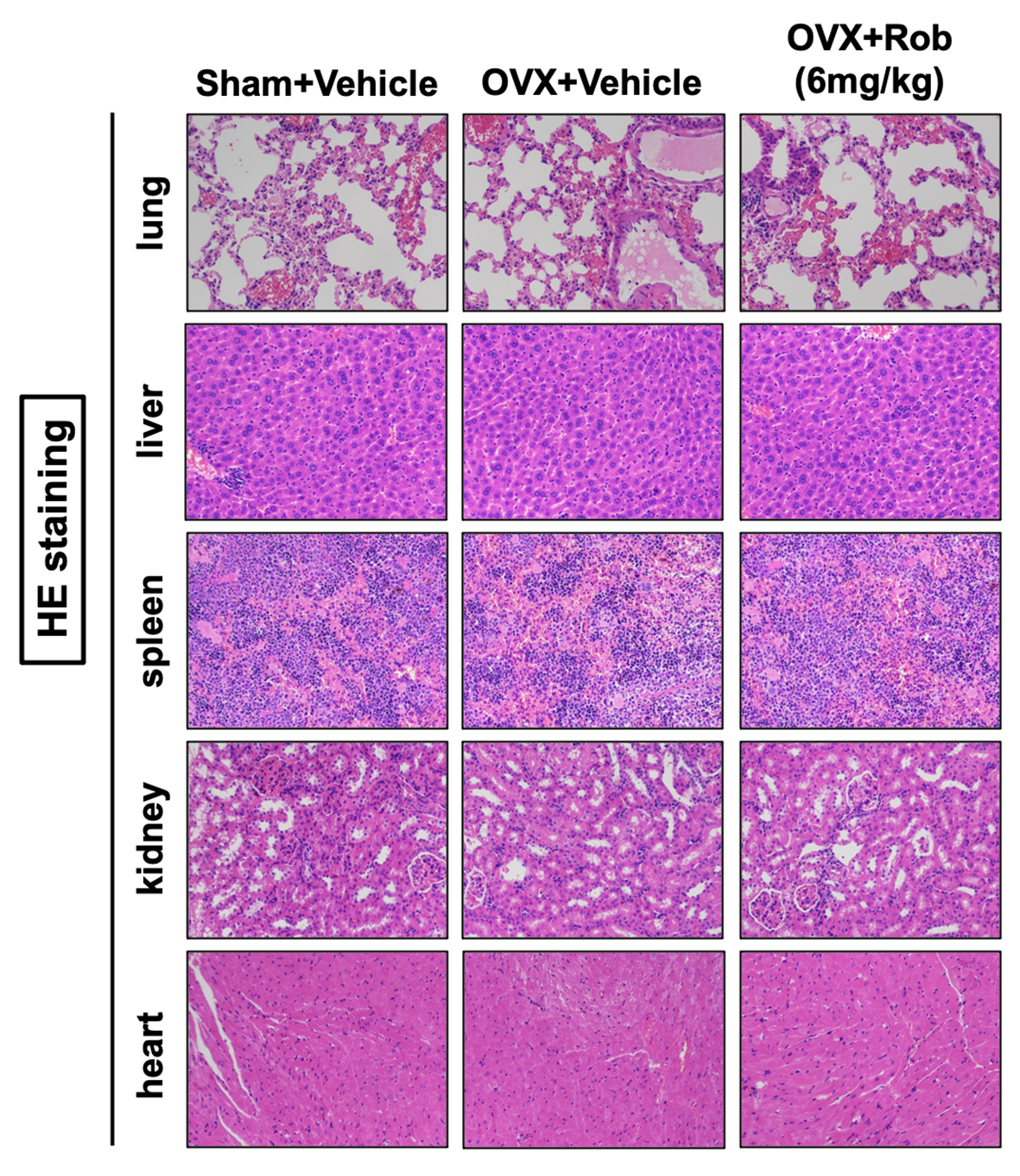

Supplement: Supplementary file 6 — FIGURE S6. Histomorphology analysis of organs of mice in each group, including lung, liver, kidney, heart, and spleen. [file CTM2-11-e392-s006.png]
