# Supplementary material for: A novel RANKL‐targeted flavonoid glycoside prevents osteoporosis through inhibiting NFATc1 and reactive oxygen species
Source: Clin Transl Med. 2021 May 21;11(5):e392. doi: 10.1002/ctm2.392 (PMC8140192; doi:10.1002/ctm2.392)
Supplement: Supplementary file 7 — Appendices [file CTM2-11-e392-s007.docx]

**Appendices**

**FIGURE S1.** The chemical structure and formula of Rob inferred from PubChem (<https://pubchem.ncbi.nlm.nih.gov>).

**FIGURE S2.** Ramachandran plot images showing the status of amino acid residues before **(A)** and after **(B)** optimization of RANKL protein structure. The stability of amino acid is demonstrated as high (red), medium (yellow) and low (white).

**FIGURE S3. (A)** Representative images of H&E staining of decalcified bone sections. Mice were treated by various doses of Rob (1 mg/kg, 3 mg/kg, and 6 mg/kg) to identify the most effective concentration for bone loss prevention. **(B-C)** Quantitative analyses of BV/TV and BS in tissue sections (n=6 per group).

**FIGURE S4.** Quantitative analysis of body-weight changes of mice in each group during Rob treatment.

**FIGURE S5.** Representative images of organs of mice in each group, including lung (A), liver (B), kidney (C), heart (D), and spleen (E). Circles one to three indicate the organs of sham group, OVX group, and OVX+Rob group, respectively.

**FIGURE S6.** Histomorphology analysis of organs of mice in each group, including lung, liver, kidney, heart, and spleen.

**TABLE S1,** Sequences of both the forward and reverse primers of mRNAs in qRT-PCR.

| **Gene** | **Forward** | **Reverse** |
| --- | --- | --- |
| *Cathepsin K* | 5’-GGGAGAAAAACCTGAAGC-3’ | 5’ -ATTCTGGGGACTCAGAGC-3’ |
| *Acp5* | 5’-TGTGGCCATCTTTATGCT-3’ | 5’-GTCATTTCTTTGGGGCTT-3’ |
| *Atp6v0d2* | 5′-GTGAGACCTTGGAAGACCTGAA-3′ | 5′-GAGAAATGTGCTCAGGGGCT-3′ |
| *Nfatc1* | 5′-CAACGCCCTGACCACCGATAG-3′ | 5′-GGCTGCCTTCCGTCTCATAGT-3′ |
| *C-fos* | 5′-GCGAGCAACTGAGAAGAC-3′ | 5′-TTGAAACCCGAGAACATC-3′ |
| *Mmp9* | 5′-CGTGTCTG GAGATTCGACTTGA-3′ | 5′-TTGGAAACTCACACGCCAGA-3′ |
| *Gapdh* | 5′-ACCACAGTCCATGCCATCAC-3′ | 5′-TCCACCACCCTGTTGCTGTA-3′ |

**TABLE S2, Antibody List**

| **Epitope/Antigen or Product name** | **Source** | **Catalog #** | **Dilution** | **Application specific details** |
| --- | --- | --- | --- | --- |
| TRAF6 | Abcam (Cambridge, MA) | ab33915 | 1: 1000 | 5% skim milk for 1 hour, overnight, 4 °C |
| NOX1 | Abcam (Cambridge, MA) | ab131088 | 1: 2000 | 5% skim milk for 1 hour, overnight, 4 °C |
| HO-1 | Abcam (Cambridge, MA) | ab68477 | 1: 1000 | 5% skim milk for 1 hour, overnight, 4 °C |
| Catalase | Abcam (Cambridge, MA) | ab76110 | 1: 1000 | 5% skim milk for 1 hour, overnight, 4 °C |
| GSR | Abcam (Cambridge, MA) | ab124995 | 1: 1000 | 5% skim milk for 1 hour, overnight, 4 °C |
| Integrin αV | Abcam (Cambridge, MA) | ab179475 | 1: 1000 | 5% skim milk for 1 hour, overnight, 4 °C |
| NFATc1 | Abcam (Cambridge, MA) | ab25916 | 1: 1000 | 5% skim milk for 1 hour, overnight, 4 °C/2% bovine serum albumin for 1 hour, overnight, 4 °C |
| Cathepsin K | Abcam (Cambridge, MA) | ab19027 | 1: 1000 | 5% skim milk for 1 hour, overnight, 4 °C |
| IkB-$\alpha$ | Abcam (Cambridge, MA) | ab32518 | 1: 1000 | 5% skim milk for 1 hour, overnight, 4 °C |
| p-ERK | Cell Signaling Technology (Beverly, MA) | 4370T | 1: 1000 | 5% skim milk for 1 hour, overnight, 4 °C |
| ERK | Cell Signaling Technology (Beverly, MA) | 4695T | 1: 1000 | 5% skim milk for 1 hour, overnight, 4 °C |
| p-p38 | Cell Signaling Technology (Beverly, MA) | 4511T | 1: 3000 | 5% skim milk for 1 hour, overnight, 4 °C |
| p38 | Cell Signaling Technology (Beverly, MA) | 8690T | 1: 1000 | 5% skim milk for 1 hour, overnight, 4 °C |
| p-JNK | Cell Signaling Technology (Beverly, MA) | 9255s | 1: 2000 | 5% skim milk for 1 hour, overnight, 4 °C |
| JNK | Cell Signaling Technology (Beverly, MA) | 9252T | 1: 1000 | 5% skim milk for 1 hour, overnight, 4 °C |
| $\beta$-actin | Beijing Biodragon Immunotechnologies (Beijing, China) | B1033 | 1: 3000 | 5% skim milk for 1 hour, overnight, 4 °C |
| NF-κB p65 | Cell Signaling Technology (Beverly, MA) | 8242T | 1:1000 | 2% bovine serum albumin for 1 hour, overnight, 4 °C |
| Dylight 488 | Biodragon (Beijing, China) | BD9002 | 1:200 | / |
| Alexa Fluor 633 | Biodragon (Beijing, China) | BD9322 | 1:200 | / |

**TABLE S3,** Analysis of clinical biochemistry of mouse blood.

| **Items** | **Sham group (n=6)** | **OVX group (n=6)** | **OVX+Rob group (n=6)** |
| --- | --- | --- | --- |
| ALB (g/dL) | 3.25$\pm$0.31 | 3.10$\pm$0.41 | 3.34$\pm$0.99 |
| ALT (U/L) | 35.12$\pm$2.36 | 36.41$\pm1$.22 | 34.82$\pm$3.17 |
| AST (U/L) | 100.32$\pm$6.45 | 95.36$\pm$7.12 | 97.12$\pm$4.33 |
| TBIL (mg/dL) | 0.31$\pm$0.10 | 0.29$\pm$0.07 | 0.33$\pm$0.11 |
| BUN (mg/dL) | 18.22$\pm$8.13 | 17.86$\pm$5.33 | 19.54$\pm$7.45 |
| CHOL (mg/dL) | 105.33$\pm$11.09 | 98.31$\pm$8.17 | 101.31$\pm$16.07 |
| CRE (mg/dL) | 0.31$\pm$0.10 | 0.39$\pm$0.05 | 0.41$\pm$0.08 |
| GGT (U/L) | 7.06$\pm$0.11 | 6.71$\pm$0.05 | 6.11$\pm$0.12 |
| GLU (mg/dL) | 276.32$\pm$21.53 | 265.16$\pm$14.11 | 299.21$\pm$29.07 |

*ALB, albumin; ALT, alanine transaminase; AST, aspartate transaminase; TBIL, total bilirubin level; BUN, blood urea nitrogen; CHOL, cholesterol; CRE, creatinine; GGT, gamma glutamyl transpeptidase; GLU, glucose.

**TABLE S4,** Hematology in the blood of mice.

| **Items** | **Sham group (n=6)** | **OVX group (n=6)** | **OVX+Rob group (n=6)** |
| --- | --- | --- | --- |
| WBC (K/$\mu$L) | 6.72$\pm$0.76 | 5.99$\pm$0.32 | 6.12$\pm$0.51 |
| RBC (M/$\mu$L) | 10.12$\pm$2.33 | 8.12$\pm$2.37 | 9.32$\pm$3.32 |
| HGB (g/dL) | 14.03$\pm$1.43 | 15.25$\pm$1.35 | 13.39$\pm$2.01 |
| HCT (%) | 47.52$\pm$11.22 | 52.32$\pm$8.34 | 53.27$\pm$10.44 |
| MCV (fL) | 50.71$\pm$3.15 | 49.61$\pm$6.22 | 49.15$\pm$3.97 |
| MCH (pg) | 14.52$\pm$1.35 | 15.01$\pm$0.76 | 14.71$\pm$0.64 |
| MCHC (g/dL) | 27.18$\pm$4.21 | 30.042$\pm$7.16 | 31.12$\pm$2.36 |
| PLT (K/$\mu$L) | 1463.12$\pm$128.56 | 1522.13$\pm$97.35 | 1525.62$\pm$155.22 |
| RDW (%) | 18.22$\pm$4.36 | 19.08$\pm$3.35 | 18.34$\pm$2.36 |
| MPV (fL) | 4.92$\pm$0.76 | 5.41$\pm$0.52 | 4.82$\pm$0.27 |
| NEUT (K/$\mu$L) | 2.02$\pm$0.05 | 1.91$\pm$0.09 | 2.12$\pm$0.07 |
| LYMPH (k/$\mu$L) | 8.22$\pm$0.85 | 7.32$\pm$1.01 | 7.52$\pm$0.73 |
| MONO (k/$\mu$L) | 0.71$\pm$0.06 | 0.59$\pm$0.05 | 0.63$\pm$0.11 |
| EO (k/$\mu$L) | 0.22$\pm$0.05 | 0.28$\pm$0.06 | 0.23$\pm$0.04 |
| BASO (mg/dL) | 0.08$\pm$0.01 | 0.05$\pm$0.00 | 0.06$\pm$0.00 |

*WBS, white blood cell count; RBC, red blood cell count; HGB, hemoglobin; HCT, hematocrit; MCV, mean corpuscular volume; MCH, mean corpuscular hemoglobin; MCHC, mean corpuscular hemoglobin concentration; PLT, platelet; RDW, red blood cell volume distribution width; PDW, platelet distribution width; MPV, mean platelet volume; NEUT, neutrophil count; LYMPH, lymphocyte count; MONO, monocyte count; EO, eosinophil count; BASO, basophil count.

**Abbreviations**

Robinin: Rob; NFATc1: nuclear factor of activated T cells 1; MAPK: mitogen-activated protein kinase; OVX: ovariectomized; BMM: bone marrow macrophages; NF-κB: nuclear factor nuclear factor kappa-light-chain-enhancer of activated B cells; RANKL: nuclear factor-κB ligand; ROS: reactive oxygen species; M-CSF: macrophage colony stimulating factor; TRAF6: TNF receptor-associated factor 6; NOX1: nicotinamide adenine dinucleotide phosphate oxidase 1; HO-1: heme oxygenase-1; PFA: paraformaldehyde; *c-fos*: proto-oncogene C-Fos; *Mmp9*: matrix metallopeptidase 9; *Atp6v0d2*: ATPase H^+^ transporting V0 subunit D2; *Acp5*: acid phosphatase 5, tartrate resistant; Rac1: Ras-related C3 botulinum toxin substrate 1; CAT: catalase; GTP: guanosine-5'-triphosphate; BV/TV: bone volume per tissue volume; Tb.Th: trabecular thickness; Tb. N: number of trabeculae; Conn.Dn: connectivity density; Oc.S/BS: osteoclast surface/bone surface; N.Oc/BS: number of osteoclasts /bone surface.
